# Localized and sustained release of Botulinum Toxin A from supramolecular peptide hydrogel for strabismus treatment

**DOI:** 10.7150/thno.128115

**Published:** 2026-05-01

**Authors:** Wei Guo, Hui Zhu, Yuchen Yao, Dan Huang, Tiantian Zhou, Xiaoqi Zhu, Jiaying Zhang, Gaolin Liang, Wenjun Zhan, Hu Liu

**Affiliations:** 1 Department of Ophthalmology, The First Affiliated Hospital with Nanjing Medical University, Nanjing 210029, China.; 2 State Key Laboratory of Digital Medical Engineering, School of Biological Science and Medical Engineering, Southeast University, Nanjing 211189, China.

**Keywords:** botulinum toxin type A, peptide, strabismus, supramolecular hydrogel, sustained release

## Abstract

**Rationale:**

Botulinum toxin type A (BTXA) is an injectable neurotoxin widely used for the nonsurgical treatment of strabismus. However, its unintended diffusion into non-target extraocular muscles often causes complications such as ptosis and reduces its therapeutic duration. Therefore, suitable delivery platforms to improve both safety and efficacy of BTXA in strabismus treatment are urgently required.

**Methods:**

In this research, we co-assembled a pentapeptide hydrogelator Nap-Phe-Phe-Lys-Lys-Lys (NapFFKKK) with BTXA under mild physiological conditions to form a supramolecular hydrogel** Gel Nap+BTXA** for localized and sustained release of BTXA. The physicochemical characteristics, cytocompatibility, and hemocompatibility of the hydrogel were evaluated *in vitro*. The *in vitro* drug release profile and *in vivo* drug diffusion characteristics were systematically evaluated using single-photon emission computed tomography (SPECT) imaging. After intramuscular injection of **Gel Nap+BTXA** into the superior rectus muscles of rabbits, the *in vivo* therapeutic efficacy and biosafety were further verified.

**Results:**

Co-assembly of BTXA with NapFFKKK resulted in the supramolecular hydrogel** Gel Nap+BTXA** with a well-organized nanofibrous network and enhanced mechanical strength, maintaining the biological activity of BTXA while enabling sustained drug release. *In vivo* experiments demonstrated that, compared with free BTXA solution, **Gel Nap+BTXA** showed about 3.1-fold higher local retention of the toxin at 24 h post-injection. Furthermore, **Gel Nap+BTXA** effectively alleviated ptosis and provided more stable and long-lasting ocular alignment correction up to 2 months, with no detectable ocular or systemic toxicity.

**Conclusions:**

This study demonstrated a safe and efficient localized delivery strategy for BTXA, offering a promising alternative for strabismus treatment.

## Introduction

Strabismus, characterized by visual axis misalignment, is a common ocular motility disorder that can lead to diplopia, suppression, and loss of binocular vision 1-4. Conventional treatment of strabismus has primarily relied on incisional surgery. However, since the approval of botulinum toxin type A (BTXA) by the Food and Drug Administration (FDA) in 1989, this injectable neurotoxic protein has provided a minimally invasive and valuable alternative for ocular alignment treatment of strabismus 5-7. The therapeutic mechanism of BTXA involves blocking the release of acetylcholine, thereby inducing temporary chemodenervation and paralysis of overactive extraocular muscles 5, 8, 9. Consequently, this reversible muscle weakening rebalances the relative muscle forces to restore ocular alignment 6, 10, 11. Compared to surgery, BTXA injection offers key advantages such as outpatient administration, tissue preservation, adjustable and repeatable correction, and a generally favorable and reversible safety profile 12-14. To date, BTXA has been successfully used in various strabismus subtypes, including infantile esotropia, acute acquired concomitant esotropia, and paralytic and restrictive strabismus, with efficacy comparable to that of conventional extraocular muscle surgery 15-18.

Despite these advantages, the injected BTXA can easily diffuse into adjacent non-target muscles, which may contribute to undesirable side effects and reducing its therapeutic duration 19-21. The most common complications include ptosis and vertical deviation 22. Clinical studies have reported that ptosis occurs in approximately 16.7-37.0% of patients and vertical strabismus in 4.0-22.0%, which could be easily blamed on poor injection techniques or because of the diffusion of BTXA 6, 17. Although these effects typically resolve within 4-8 weeks, in severe cases they may persist for up to three months, posing a risk for amblyopia in children and anxiety for patients and caregivers 12, 23. Moreover, diffusion of BTXA away from target muscle reduces its therapeutic efficacy, requiring higher doses or repeated injections for expected therapeutic effect 14, 18. Such regimens not only increase patient discomfort and treatment cost, but may also lead to biosafety issues such as cumulative local tissue injury or fibrosis. Therefore, developing suitable delivery vehicles to overcome these off-target diffusion-related issues of BTXA is urgently needed.

To achieve the abovementioned goal, supramolecular peptide hydrogels represent an attractive platform owing to their facile synthesis, tunable molecular design, good biodegradability, and excellent biocompatibility 24-27. Peptide hydrogels are typically formed through reversible non-covalent interactions, such as hydrogen bonding, π–π stacking, and electrostatic forces, to generate hydrated, three-dimensional networks that mimic the extracellular matrix (ECM) 28-31. Remarkably, the low-molecular-weight peptide hydrogel networks can physically capture protein drugs and prevent their irreversible aggregation, thereby achieving both effective loading and functional retention 32, 33. Moreover, their inherent injectability, reversible responsiveness, and ECM-like softness make them especially suitable for localized ocular drug delivery 34, 35, and their ECM-like structural network helps stabilize injected agents within the local tissue microenvironment 36, 37. Additionally, peptide hydrogels can achieve increased retention and sustained release of drugs in ophthalmic applications 38-41. In view of these advantages, we naturally think of using peptide hydrogels as a controlled delivery matrix for BTXA in extraocular muscle injection, thereby achieving enhanced safety and efficacy of BTXA in strabismus treatment. However, to the best of our knowledge, such supramolecular peptide hydrogel delivery system has not been reported to date.

**Figure 1:**
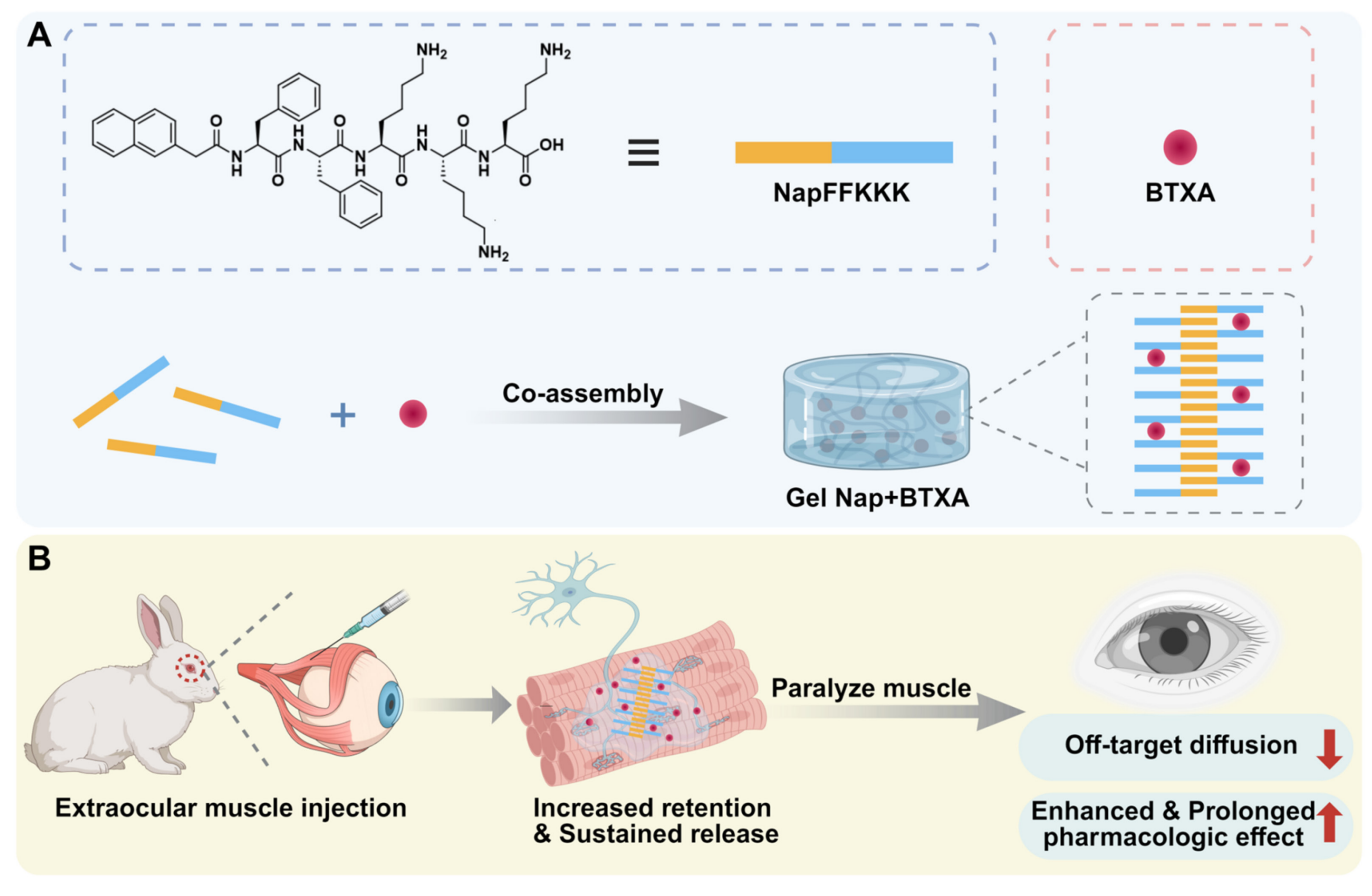
** Schematic illustration of Gel Nap+BTXA for strabismus treatment.** (A) Co-assembly of NapFFKKK with BTXA to form **Gel Nap+BTXA**. (B) Illustration of local delivery of **Gel Nap+BTXA** for BTXA release in extraocular muscle to provide a safer and more effective approach for strabismus treatment.

Herein, we intend to employ a supramolecular peptide hydrogel to achieve localized and sustained release of BTXA for strabismus treatment (**Figure 1**). For this purpose, a pentapeptide hydrogelator Nap-Phe-Phe-Lys-Lys-Lys (NapFFKKK) was chosen to co-assemble with BTXA to facilely prepare a supramolecular hydrogel **Gel Nap+BTXA** (**Figure 1A**). We hypothesize that this biocompatible supramolecular hydrogel can achieve increased retention and sustained release of BTXA at the extraocular muscle injection site, thereby enhancing its alignment efficacy while minimizing adverse effects (**Figure 1B**). In this work, we systematically evaluated the characterization, *in vitro* biocompatibility, *in vitro* drug release behavior, and *in vivo* drug diffusion, therapeutic efficiency and biosafety of this delivery platform. Our findings provide the first evidence of peptide hydrogel-mediated BTXA delivery in extraocular muscles, proposing a safer and more effective pharmacologic approach for the management of strabismus.

## Materials and Methods

### Preparation of the hydrogels

To prepare **Gel Nap**, the peptide hydrogelator NapFFKKK was first dissolved in phosphate-buffered saline (PBS, pH = 6.5) under vortex mixing to obtain a 1.0 wt% solution. The pH was subsequently adjusted to 7.8 using sodium carbonate (Na_2_CO_3_), and the solution was left at room temperature for 2 h to allow self-assembly into a stable hydrogel. For the drug-loaded formulation, BTXA solution was mixed with the peptide solution to achieve a final toxin concentration of 50 U/mL and gently mixed to ensure uniform distribution. The pH was then adjusted to 7.8 to induce gelation at room temperature.

### Characterization of the hydrogels

For transmission electron microscopy (TEM), dispersions of hydrogel were dropped onto carbon-coated copper grids. After removing the excess liquid, the grids were air-dried at room temperature prior to observation. Rheology characterization was carried out using a parallel-plate configuration to evaluate the viscoelastic behavior of hydrogels. Measurements of frequency sweep were performed at strain of 1% across 0.1-10.0 Hz, and strain sweeps were carried out at 1.0 Hz with strain ranging from 0.1% to 10%.

### Culture and identification of human extraocular muscle fibroblasts

Primary human extraocular muscle fibroblasts were obtained from excised muscle tissues of patients undergoing strabismus surgery, with ethical approval and informed patient consent. The primary cell cultures were established using the tissue explant method. Briefly, the muscle samples were sectioned into small pieces and placed in culture dishes to facilitate cell migration and adhesion. Within 3-7 days, fibroblast outgrowth was noted around the tissue pieces, and significant cell spreading was observed by approximately 10 days. After removing the residual tissue fragments, cells were cultured in DMEM containing 20% fetal bovine serum (FBS) and 1% penicillin-streptomycin, kept at 37 ℃ in a humidified incubator with 5% CO_2_. Cells at 3-6 passages were used for subsequent experiments. For cell identification, fibroblasts were stained for vimentin following the manufacturer’s instructions with a DAB detection kit. After DAB color development and hematoxylin counterstaining, a light microscope was used to observe cells to verify typical fibroblast morphology and vimentin positivity.

### Radiolabeling of BTXA with ^131^I

BTXA was radiolabeled with ^131^I using the Iodogen oxidation method. A vial pre-coated with Iodogen (200 µg) was used to catalyze the labeling reaction. One vial of BTXA (100 U) was dissolved in 600 µL ultrapure water, and 200 µL of this solution was mixed with ^131^I (approximately 2.5 mCi). The reaction was carried out at room temperature for 5-6 min. To verify labeling, thin-layer chromatography (TLC) was performed using 10 mM citric acid as the mobile phase, and the plate was scanned to confirm the formation of ^131^I-labeled BTXA (^131^I-BTXA) and the removal of unbound ^131^I for subsequent release and diffusion analyses.

### *In vitro* release of ^131^I-BTXA from hydrogels

For the release study, **Gel Nap+^131^I-BTXA** was prepared by co-assembly of NapFFKKK (1.0 wt%) with ^131^I-BTXA under the same conditions as described above. Subsequently, 2 mL of PBS (pH 7.4) was gently added onto 200 µL of the hydrogel and incubated at 37 ℃. One-tenth of the supernatant was withdrawn and replaced with the same volume of fresh PBS at set times. The radioactivity (counts per minute, CPM) of each collected sample was measured using a γ-counter, and the cumulative release of ^131^I-BTXA was calculated based on the ratio of released activity relative to the total loaded activity.

### Animal preparation and administration

Male New Zealand white rabbits aged 16 weeks, weighing 3.0-3.5 kg were used for the* in vivo* experiments. All animals received general and topical ocular anesthesia, and underwent intramuscular injection in the right eye. A 29-gauge needle was inserted through the conjunctiva into the superior rectus muscle at 5 mm posterior to the insertion and advanced 5-10 mm parallel to the muscle fibers. 100 µL of different sample solution were then slowly injected into the muscle. For the treatment groups, the injected dose contained 5 U BTXA.

### *In vivo* diffusion evaluation

To assess the *in vivo* diffusion of BTXA, rabbits were anesthetized and received intramuscular injections into the right superior rectus muscle using three formulations: ^131^I-BTXA, **Gel Nap+^131^I-BTXA**, and free ^131^I. Single-photon emission computed tomography (SPECT) images were acquired at 0, 1, 2, 4, 6, 16, and 24 h post-injection to monitor the local retention and diffusion of ^131^I-BTXA. The acquired planar images were analyzed using PMOD software. Circular regions of interest (ROIs) were manually drawn over the injection site to quantify local radioactivity. The ROI counts were extracted and normalized to the initial counts at 0 h to obtain relative retention (% of 0 h). The averaged ROI values from three rabbits in each group were used for quantitative analysis.

### Evaluation of ptosis

To evaluate post-injection ptosis induced by toxin diffusion, photographs of the injected eye were taken at 7, 14, 30, and 60 days after injection under consistent environmental and lighting conditions. The palpebral fissure height was defined as H_1_ for ptosis assessment. ΔH_1_ was calculated by subtracting the post-injection value from the pre-injection value to reflect the extent of toxin diffusion in different groups. A larger ΔH_1_ indicated a greater degree of ptosis, indicating more severe toxin diffusion. Measurements were performed using ImageJ software, with three photographs analyzed for each rabbit and each image measured three times to obtain the average value.

### Evaluation of ocular alignment

The photographs collected for ptosis evaluation were further analyzed to assess ocular alignment correction. The perpendicular distance from the pupil center to the lower eyelid margin was defined as H_2_ and ΔH_2_ was calculated by subtracting the post-injection value from the pre-injection value to evaluate alteration of ocular deviation. A greater ΔH_2_ indicated a stronger pharmacologic effect on ocular alignment. Similarly, all measurements were performed using ImageJ software, with three photographs analyzed per rabbit and each image measured three times calculate the average value.

### Histological evaluation of muscle morphology and biosafety

The superior rectus muscle samples were collected at 7, 14, 30, and 60 days after injection. Segment of muscle tissue around the injection site was excised, fixed and embedded. The sections were stained with hematoxylin and eosin (H&E) according to standard protocols. Representative regions from the orbital and global layers were selected for quantitative analysis. The cross-sectional area (CSA) of individual muscle fiber was measured using ImageJ software, with at least three randomly selected fields analyzed per section and a minimum of 100 fibers quantified for each sample. The mean myofiber CSA for each rabbit was calculated and used for statistical comparison among groups at each time point. To evaluate local tissue compatibility, additional muscle samples were collected at 7 and 30 days after injection and processed for paraffin embedding and 5-μm sectioning. Collagen deposition and fibrosis were evaluated using Masson’s trichrome staining.

### Intraocular pressure measurement

Intraocular pressure (IOP) was measured with a handheld rebound tonometer to assess potential effects on ocular physiology. IOP of right eyes was recorded before injection and at 1, 2, 3, and 7 days after injection. Each measurement was performed three times to calculate the mean value for analysis.

### Blood biochemical analysis

To assess systemic biosafety, rabbit blood samples were collected from the auricular vein at 7 days after injection. Serum was obtained by centrifugation at 3000 rpm for 10 min, and the levels of biochemical parameters including alanine aminotransferase (ALT), aspartate aminotransferase (AST), urea nitrogen (BUN), creatinine (CREA), creatine kinase (CK), and lactate dehydrogenase (LDH) were analyzed.

### Statistical analysis

All data were presented as mean ± standard deviation (SD). Differences among multiple groups were assessed using one-way or two-way analysis of variance (ANOVA) and pairwise comparisons were performed with two-tailed t tests. A *p* value < 0.05 was considered statistically significant (*p < 0.05, **p < 0.01, ***p < 0.001). GraphPad Prism 10.0 was used for statistical analysis.

## Results and Discussion

### Preparations and characterizations of the hydrogels

**Figure 2:**
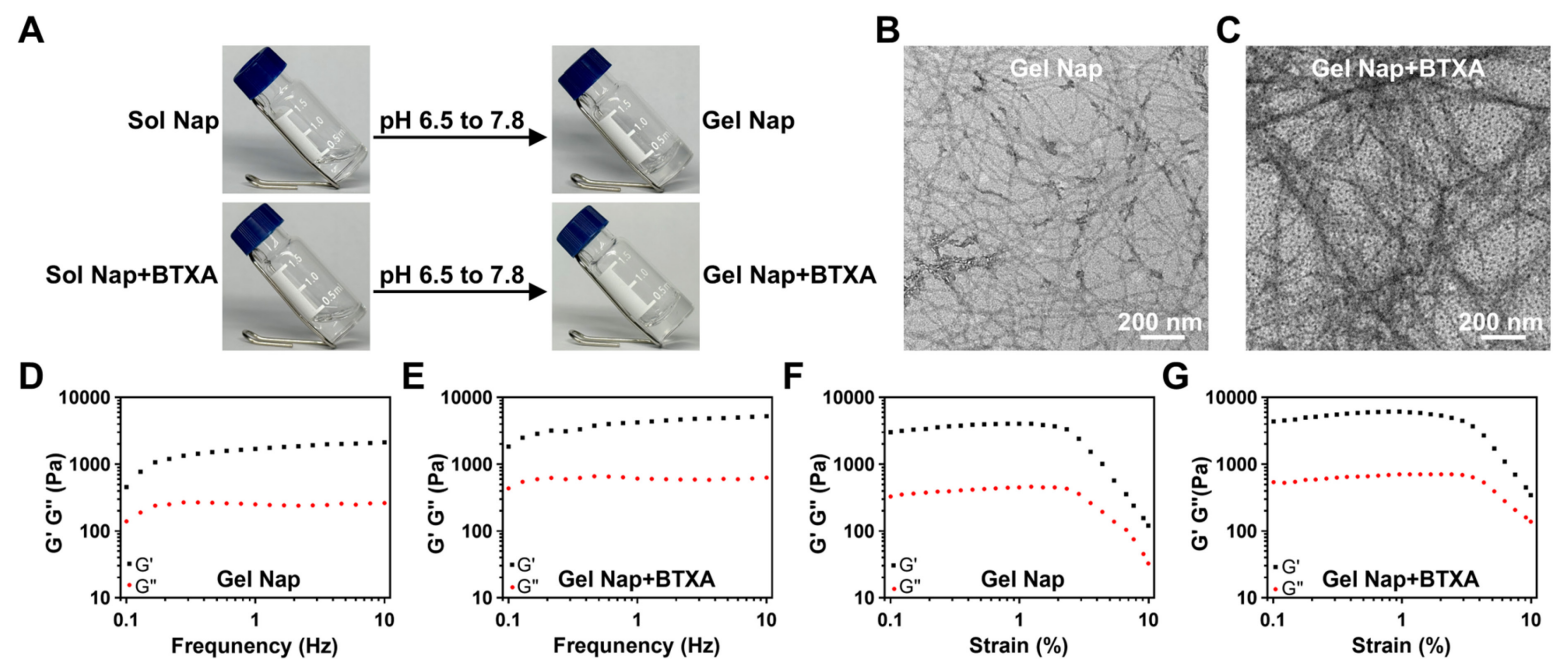
**Characterizations of Gel Nap** and** Gel Nap+BTXA.** (A) Sol-Gel transition of NapFFKKK and NapFFKKK+BTXA after pH adjustment. (B-C) TEM images of **Gel Nap** and **Gel Nap+BTXA**, respectively. Scale bar: 200 nm. (D-G) Frequency (γ = 1%) and strain (f = 1 Hz)-dependent rheology of **Gel Nap** (D, F) and **Gel Nap+BTXA** (E, G).

We began the study with the synthesis of NapFFKKK using standard solid-phase peptide synthesis (**Scheme S1**) 42. The peptide hydrogelator was purified by high-performance liquid chromatography (HPLC) and characterized with electrospray ionization mass spectrometry (ESI-MS) and nuclear magnetic resonance (NMR) spectroscopy (**Figures S1-S3**). Next, we investigated the hydrogelation property of NapFFKKK. Systematic formulation screening was performed by varying peptide concentration (0.5, 1.0, and 2.0 wt%) and gelation pH values (6.5, 7.4, and 7.8). Based on the integrated optimization of gel structure and mechanical properties, a formulation consisting of 1.0 wt% peptide and a pH value of 7.8 was selected (**Figures S4-S5**). Briefly, 2.5 mg of NapFFKKK powder was dissolved in 250 μL of PBS (10mM, pH 6.5) to obtain a 1.0 wt% solution. After adjusting the pH to 7.8 with Na_2_CO_3_ and incubating for 2 h at room temperature, the mixture formed a stable and transparent hydrogel (i.e., **Gel Nap,** top row of **Figure 2A**). To reveal the hydrogelation mechanism, the critical aggregation concentrations (CACs) of NapFFKKK at pH 6.5 and pH 7.8 were measured by ultraviolet-visible transmittance analysis. As shown in **Figure S6**, the CAC value of NapFFKKK at pH 6.5 was 354.8 μM and that at pH 7.8 was 187.7 μM, which was attributed to the pH-induced deprotonation of the amino groups in the hydrogelator. Substantial variation in these two CAC values suggested that NapFFKKK underwent pH-triggered self-assembly to form **Gel Nap** at the above condition. In addition, **Gel Nap** maintained overall structural integrity in both PBS (pH 7.4) and 10% FBS for up to 48 h (**Figure S7**). Further swelling analysis showed that **Gel Nap** remained a newly constant swelling ratio after 48 h of incubation with PBS (pH 7.4) (**Figure S8**). These results confirmed the excellent stability of **Gel Nap** under physiological conditions.

Furthermore, the co-assembly potential between BTXA and NapFFKKK was investigated. Specifically, BTXA was added to the peptide solution (1.0 wt%) in PBS (10 mM, pH 6.5) and gently mixed to ensure uniform distribution. Based on the clinically used concentration of BTXA for therapeutic injection 7, 43, 44, the drug concentration was set at 50 U/mL. Following the same pH adjustment and incubation process, the mixture also successfully formed a stable hydrogel (i.e., **Gel Nap+BTXA**, bottom row of **Figure 2A**). Compared with lower or higher BTXA concentrations, the 50 U/mL formulation exhibited denser nanofibers and higher rheological moduli, indicating a more mechanically robust hydrogel network (**Figure S9**). To further verify whether hydrogel encapsulation would impair the biological activity of BTXA, we performed a Western blot analysis to assess the cleavage of SNAP-25 protein in Neuro-2a cells treated with PBS, free BTXA, or **Gel Nap+BTXA**. Compared with the PBS group, the SNAP-25 band intensity was reduced in both BTXA and **Gel Nap+BTXA** groups, while no significant difference was observed between the two treatment groups (**Figure S10**). These results suggested that this mild encapsulation strategy could effectively preserve the bioactivity of the protein drug BTXA throughout hydrogel formation 45.

Subsequently, we used TEM to evaluate the effect of BTXA incorporation on the hydrogel network. The results showed that **Gel Nap** consisted of uniform nanofibers with an average diameter of 11.0 ± 2.4 nm. Interestingly, **Gel Nap+BTXA** exhibited denser and more entangled nanofibers with a significantly larger mean diameter of 18.3 ± 3.6 nm (**Figure 2B-C** and **Figure S11).** The above phenomena indicated that BTXA incorporation did not disrupt peptide self-assembly, and might enhance intermolecular interactions through additional non-covalent associations. Furthermore, the rheological properties of **Gel Nap** and **Gel Nap+BTXA** were tested. The results in **Figure 2D-G** confirmed the viscoelastic nature of both hydrogels, as the storage modulus (G′) remained higher than the loss modulus (G″) throughout the examined frequency (0.1-10 Hz) and strain (0.1-10%) ranges. Additionally, the G′ and G″ values of **Gel Nap+BTXA** were higher than those of** Gel Nap**, indicating that BTXA incorporation increased the viscoelasticity and structural stability of the hydrogel. Collectively, these results demonstrated that the protein drug BTXA could co-assemble with NapFFKKK to form a supramolecular hydrogel with enhanced mechanical strength.

### *In vitro* biocompatibility assessment of Gel Nap

**Figure 3:**
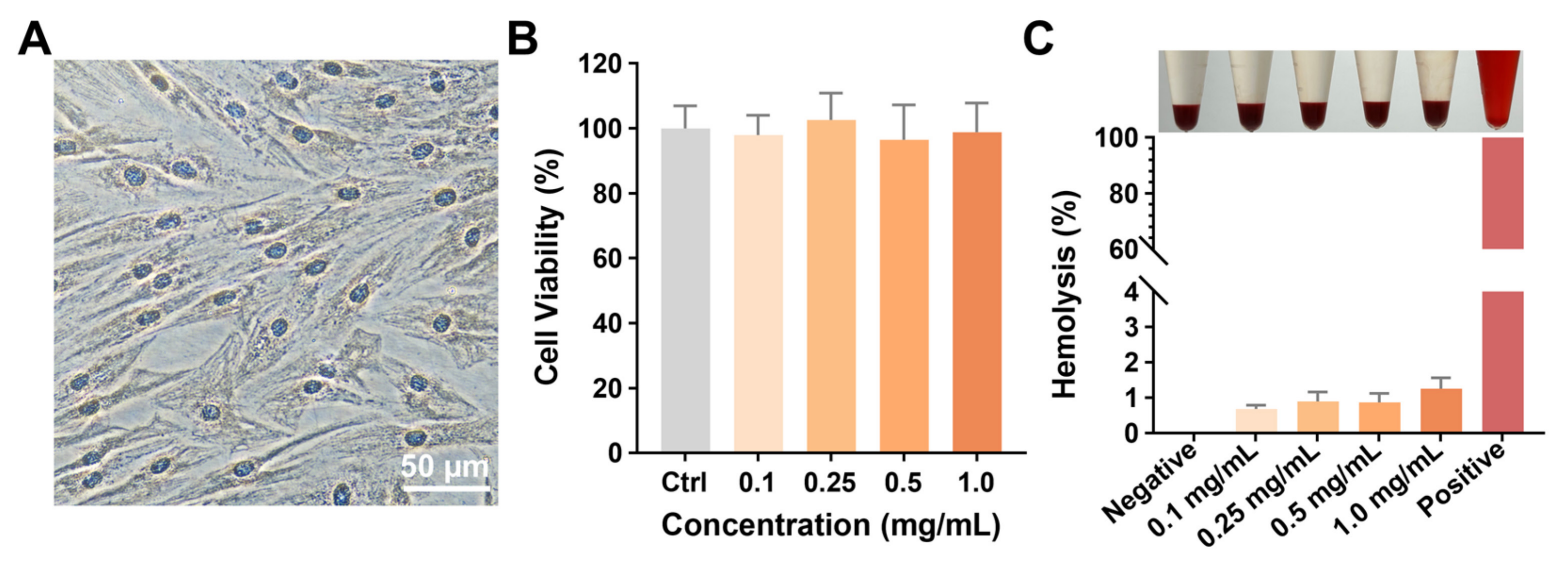
** Cytocompatibility and hemocompatibility of Gel Nap.** (A) Immunohistochemical staining for vimentin (brown, DAB) with hematoxylin counterstain (blue) of human extraocular muscle fibroblasts. Scale bar: 50 µm. (B) Cell viability of extraocular muscle fibroblasts treated with 0.1-1.0 mg/mL **Gel Nap** for 24 h (mean ± SD, *n* = 3). (C) Hemolysis of rabbit erythrocyte treated with 0.1-1.0 mg/mL **Gel Nap** (mean ± SD, *n* = 3).

The *in vitro* biocompatibility of** Gel Nap** was assessed after the preparation and characterization of the hydrogels. First, we successfully cultured primary human extraocular muscle fibroblasts (**Figure 3A**) and performed a CCK-8 assay to evaluate the cytocompatibility of **Gel Nap**. The results showed that cells maintained high viability (> 90%) after 24 h of incubation with hydrogel at concentrations ranging from 0.1 to 1.0 mg/mL, suggesting the good cytocompatibility of **Gel Nap** (**Figure 3B**). Flow cytometric analyses echoed the above results, suggesting the high cytocompatibility of **Gel Nap** (**Figure S12**). Subsequently, a hemolysis test was conducted to determine the blood compatibility of **Gel Nap**. At all tested concentrations, **Gel Nap** caused less than 5% hemolysis, which was markedly lower than that of the positive control (1% Triton X-100) and close to the baseline level of the PBS group (**Figure 3C**). Collectively, these results confirmed that **Gel Nap** possessed excellent cytocompatibility and hemocompatibility, indicating its potential as a safe and biocompatible carrier for localized BTXA delivery.

### *In vitro* cumulative release and* in vivo* diffusion of BTXA

**Figure 4:**
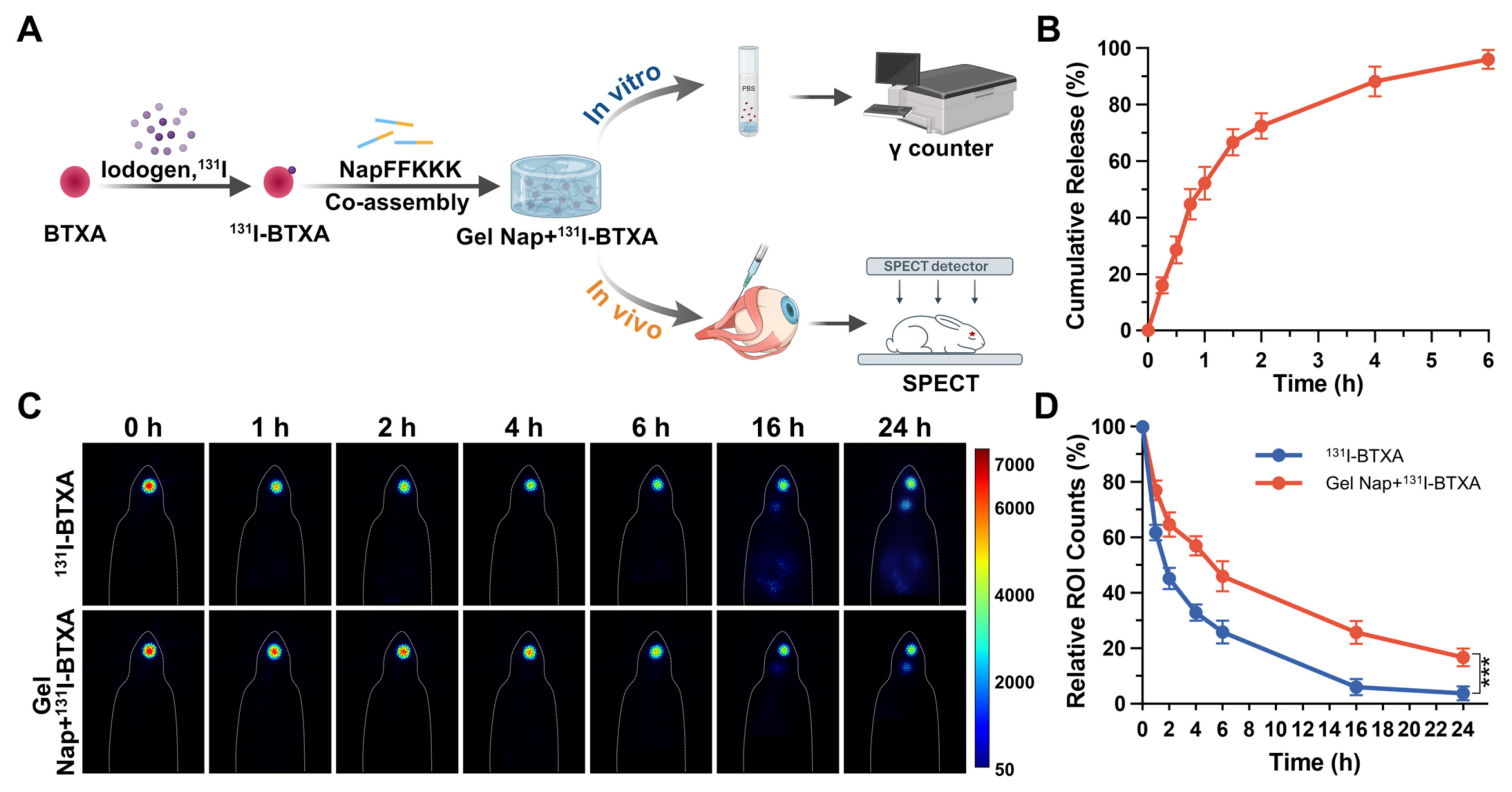
***In vitro* cumulative release and *in vivo* evaluation of BTXA diffusion.** (A) Schematic illustration of *in vitro* release and *in vivo* evaluation of diffusion. (B) *In vitro* cumulative release of ^131^I-BTXA from **Gel Nap+^131^I-BTXA**. (C) SPECT images at different time points after intramuscular injection of ^131^I-BTXA or **Gel Nap+^131^I-BTXA**. (D) Quantitative analysis of relative ROI counts between ^131^I-BTXA and **Gel Nap+^131^I-BTXA** group (mean ± SD, *n* = 3, ***p < 0.001).

To visualize and quantify its release and diffusion behavior, BTXA was first radiolabeled with ^131^I under mild aqueous conditions. TLC analysis confirmed the successful ^131^I radiolabeling of BTXA, as evidenced by a single major peak corresponding to ^131^I-BTXA (**Figures S13-S14**). The radiolabeled proteins were then co-assembled with NapFFKKK to form **Gel Nap+^131^I-BTXA**, and the *in vitro* drug release behavior of the hydrogel was subsequently evaluated by a γ counter (**Figure 4A**). The results showed a rapid release of ^131^I-BTXA from **Gel Nap+^131^I-BTXA**, reaching about 60% within 1.5 h, followed by a slower release phase, with cumulative release exceeding 90% at 5 h (**Figure 4B**). Given that supramolecular peptide hydrogels help maintain protein activity 34, such a sustained ^131^I-BTXA release behavior ensures that most toxin remains bioactive.

Following the *in vitro* release characterization, SPECT imaging was performed to evaluate the *in vivo* retention and diffusion of BTXA. In the absence of a standardized strabismus animal model, the superior rectus muscle is commonly selected in ocular motility and structural remodeling studies because of its accessibility and well-defined anatomical orientation 46, 47. Given that injection technique may influence BTXA distribution 48, 49, all injections were performed by an experienced ophthalmologist. To empirically exclude the potential influence of injection technique, we further performed an additional fluorescence-based validation experiment in which BTXA was co-injected with FITC-Dextran of comparable molecular weight (150 kDa). *Ex vivo* fluorescence imaging confirmed accurate and reproducible intramuscular injection into the superior rectus muscle (**Figure S15**). In a preliminary experiment, free ^131^I was injected into the right superior rectus muscle of each healthy New Zealand rabbit, and widespread systemic distribution was observed at 1 h (**Figure S16**). Subsequently, the rabbits were randomly divided into two groups (*n* = 3), and underwent either ^131^I-BTXA solution or **Gel Nap+^131^I-BTXA** (both containing 5 U BTXA) injection into the right superior rectus muscle. Time-course SPECT images showed that, compared with the BTXA group, the **Gel Nap+^131^I-BTXA** group exhibited a stronger and more localized radioactivity signal at the injection site during the tested release period (**Figure 4C**). Quantitatively, at 24 h, the radioactivity signal in the **Gel Nap+^131^I-BTXA** group was 2.7-fold higher than that in the BTXA group, indicating that hydrogel delivery effectively enhanced drug retention in the injection site (**Figure 4D**). Moreover, at 24 h post-injection, we excised the superior rectus muscles and non-target extraocular muscles and measured their radioactivity (**Table S1**). Of note, the superior rectus muscle radioactivity in the **Gel Nap+^131^I-BTXA** group was 3.1-fold higher than that in the ^131^I-BTXA group. In contrast, radioactivity levels in non-target muscles were consistently higher in the ^131^I-BTXA group. This was particularly evident in the levator palpebrae superioris and lateral rectus muscles, where values were significantly higher than in the **Gel Nap+^131^I-BTXA** group. Notably,* in vivo* BTXA retention lasted substantially longer than the* in vitro* release period. This difference was due to the different release environments in *in vivo* and *in vitro* experiments, as well as the additional retention time of the released BTXA at the injection site. Taken together, these results indicated that **Gel Nap+^131^I-BTXA** promoted local retention of BTXA at the extraocular muscle injection site, thereby minimizing off-target diffusion *in vivo*.

### Evaluation of *in vivo* therapeutic effect

**Figure 5:**
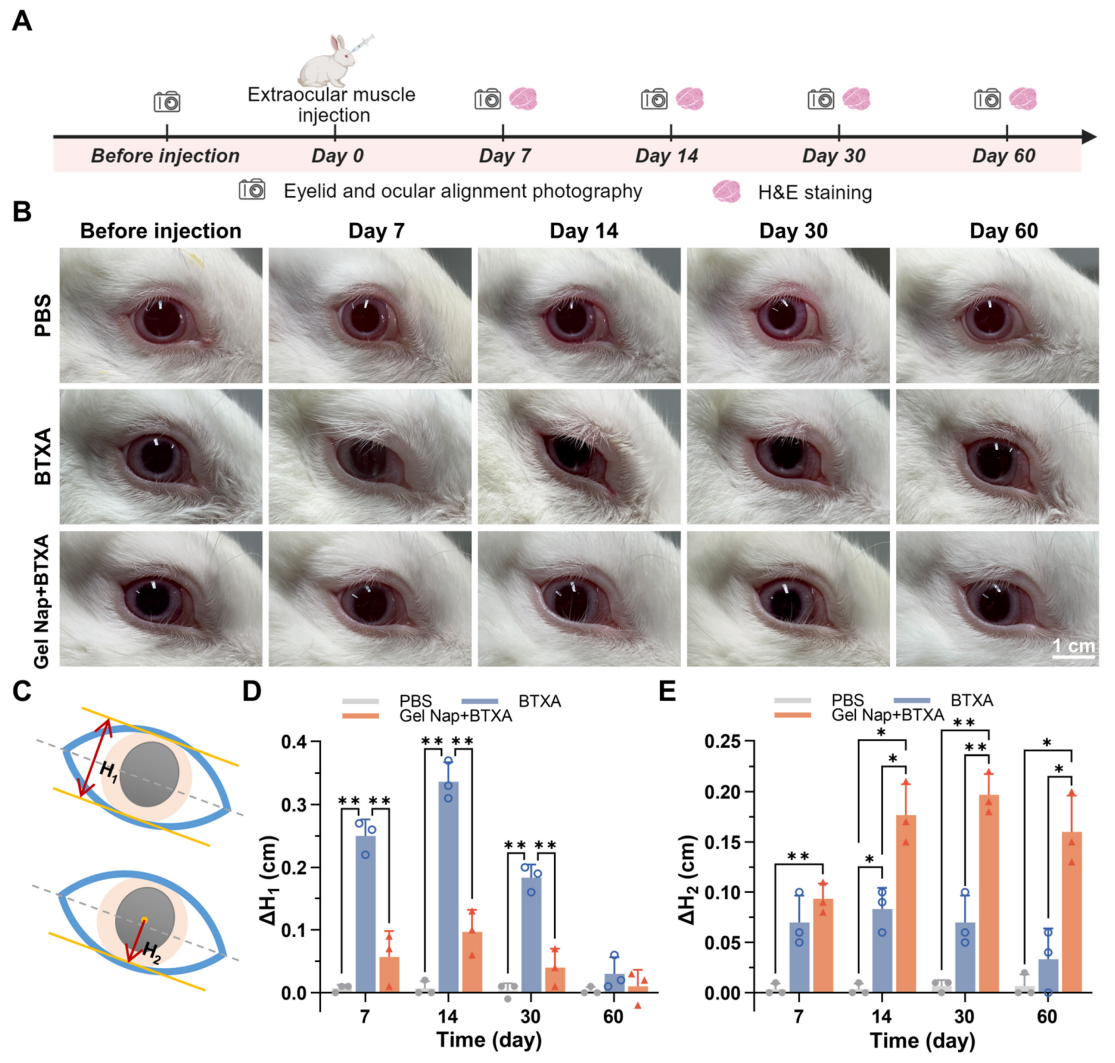
*** In vivo* assessment of ptosis and ocular alignment.** (A) Experimental schedule for *in vivo* therapeutic effect. (B) Representative photographs of the injection eye in the PBS, BTXA, and **Gel Nap+BTXA** groups at 7, 14, 30, and 60 days after injection. Scale bar: 1 cm. (C) Illustrations of ptosis measurement (H_1_) and ocular deviation measurement (H_2_). (D) Quantitative analysis of ΔH_1_ in the three groups (mean ± SD, *n* = 3, *p < 0.05, **p < 0.01). (E) Quantitative analysis of ΔH_2_ in the three groups. (mean ± SD, *n* = 3, *p < 0.05, **p < 0.01).

Encouraged by the above positive results, we further evaluated the *in vivo* therapeutic efficacy of **Gel Nap+BTXA** (**Figure 5A**). New Zealand rabbits were randomly allocated to three groups (*n* = 3) and underwent different intramuscular injections into the right superior rectus muscle as follows: PBS, BTXA, or **Gel Nap+BTXA**. Ptosis is a common side effect after extraocular muscle injection of BTXA, caused by unintended diffusion of the toxin to the levator palpebrae superioris muscle. To evaluate whether hydrogel encapsulation could effectively reduce this adverse side effect, photographs were taken at 7, 14, 30, and 60 days after injection. The results revealed a transient eyelid droop in both BTXA and **Gel Nap+BTXA** groups during the early post-injection phase (**Figure 5B**). However, this adverse effect was more severe and lasted longer in the BTXA group. Furthermore, the degree of ptosis was quantified by measuring the palpebral fissure height (H_1_, top row of **Figure 5C**) before and after different treatments. Quantitative analysis of ΔH_1_ demonstrated that the BTXA group exhibited a marked increase at day 7 and day 14, followed by a gradual recovery by 60 days (**Figure 5D**). In contrast, **Gel Nap+BTXA** produced smaller ΔH_1_ values at all time points, and the palpebral fissure height nearly returned to baseline within 30 days. These findings indicated that hydrogel encapsulation significantly reduced both the extent and duration of ptosis by restricting toxin diffusion beyond the target muscle. Furthermore, ocular alignment was assessed to verify the enhanced therapeutic effect of **Gel Nap+BTXA*** in vivo*. Specifically, the deviation of the treated eye was quantified by measuring the distance from the pupil center to lower eyelid margin (H_2_, bottom row of **Figure 5C**), and ΔH_2_ was calculated for quantitative analysis. As shown in **Figure 5E**, the **Gel Nap+BTXA** group exhibited greater ocular alignment alteration than the BTXA group at each time point post-injection. Of note, these differences were particularly pronounced in the middle and later stages (14, 30, and 60 days). Similar trends were also observed in an additional low-dose experiment using 2.5 U BTXA, in which **Gel Nap+BTXA**-mediated delivery still showed reduced ptosis and improved ocular alignment correction (**Figure S17**). These results suggested that **Gel Nap+BTXA** could effectively enhanced and prolonged the therapeutic effect of BTXA in strabismus treatment.

**Figure 6:**
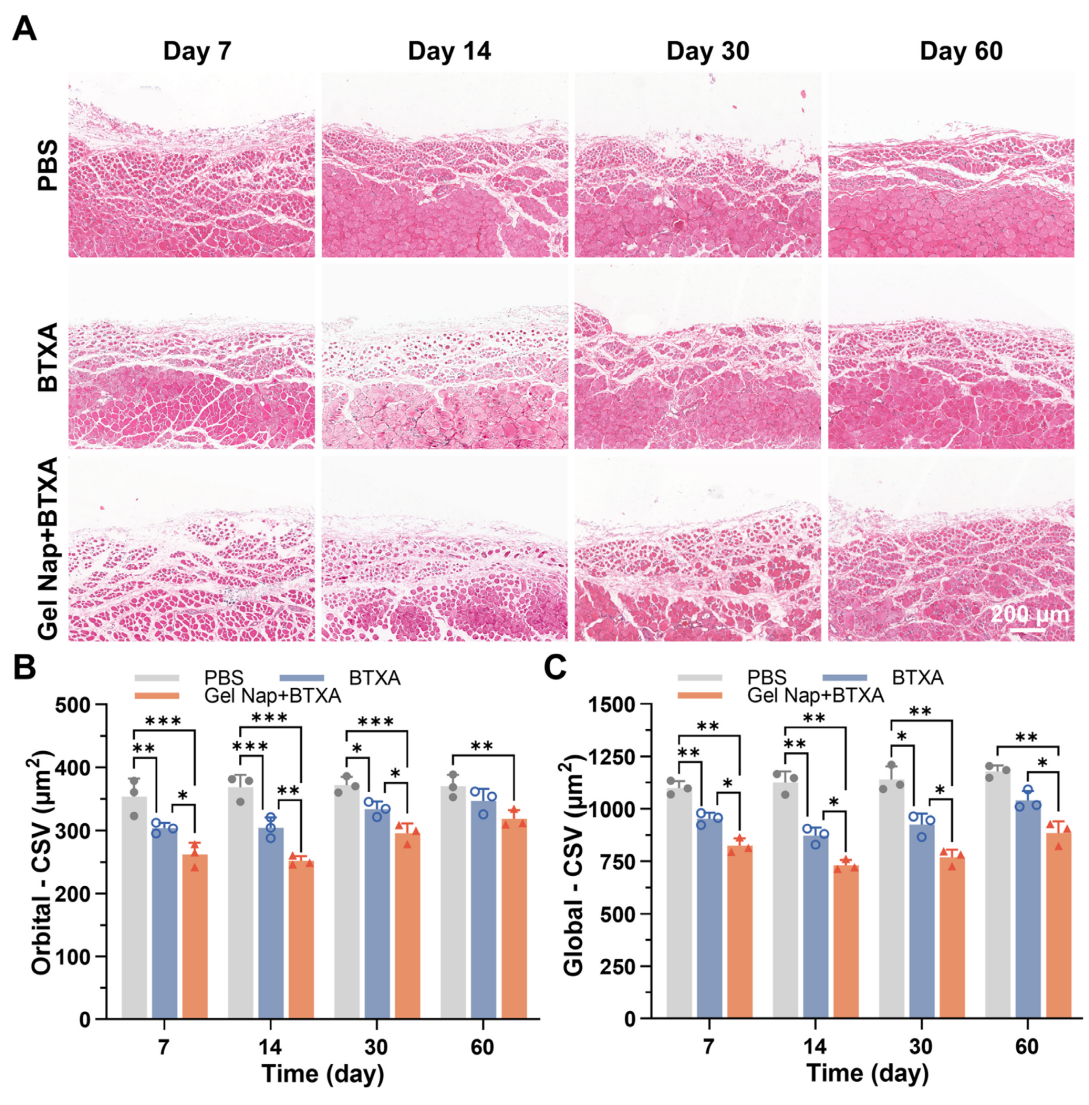
**Histological evaluation of extraocular muscle fiber cross-sectional area (CSA)**. (A) Representative H&E staining images of superior rectus muscles in the PBS, BTXA, and **Gel Nap+BTXA** groups at 7, 14, 30, and 60 days after injection. Scale bar: 200 µm. (B) Quantitative analysis of muscle fiber CSA in the orbital layer. (C) Quantitative analysis of muscle fiber CSA in the global layer (mean ± SD, *n* = 3, *p < 0.05, **p < 0.01, ***p < 0.001).

Previous studies have shown that intramuscular injection of BTXA can induce muscle fiber atrophy, accompanied by structural remodeling and partial fibrosis 50. To further investigate the effects of **Gel Nap+BTXA*** in vivo*, H&E staining was performed on muscle sections collected at 7, 14, 30, and 60 days after injection. As displayed in **Figure 6A**, the muscle fibers in the PBS group exhibited a regular morphology with a tightly packed arrangement. In contrast, both the BTXA and **Gel Nap+BTXA** groups showed evident myofiber atrophy and increased inter-fiber spacing at early stages. Despite gradual morphological recovery over time, the structural alterations in the **Gel Nap+BTXA** group persisted for a longer duration, suggesting that the hydrogel enabled more sustained local action of BTXA on the target muscle and thereby prolonged its pharmacological effect (**Figure 6A**). Given the intrinsic architectural differences between the orbital and global layers of extraocular muscles 51, the CSA of muscle fibers in these two layers were analyzed separately. At all examined time points, the CSA in the **Gel Nap+BTXA** group was significantly smaller than that of the BTXA group (**Figure 6B**). Remarkably, the CSA of the BTXA group had nearly returned to baseline at 2 months post-injection, whereas that of the **Gel Nap+BTXA** group remained significantly smaller than in the PBS group (**Figure 6B**). A similar trend was observed in the global layer of the extraocular muscle (**Figure 6C**). These results collectively suggested that the hydrogel-mediated BTXA delivery induced a stronger and longer-lasting modulation of extraocular muscle morphology, potentially achieving equivalent therapeutic efficacy with lower drug doses and fewer injections.

To further explore the potential molecular basis underlying the enhanced and prolonged therapeutic effects of hydrogel-mediated BTXA delivery, transcriptomic profiling of treated extraocular muscles was performed. Differential expression and Gene ontology enrichment analysis indicated that altered genes were predominantly enriched in biological processes related to extracellular matrix organization, collagen metabolism, and cell adhesion (**Figure S18**). These processes are closely associated with muscle structural remodeling, and may contribute to the enhanced and prolonged functional modulation observed following **Gel Nap+BTXA** treatment.

### *In vivo* biosafety evaluation

**Figure 7:**
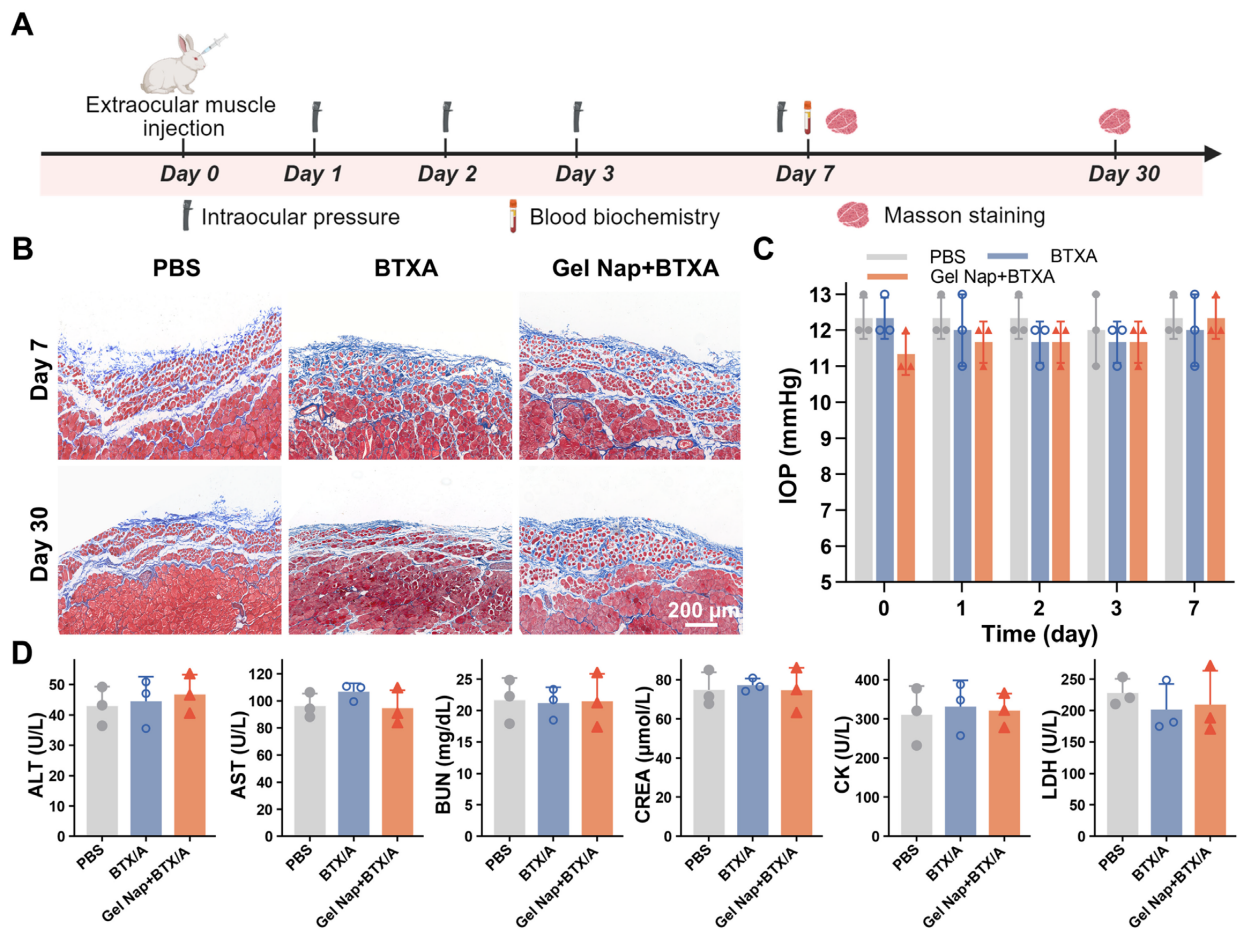
***In vivo* biosafety evaluation of Gel Nap+BTXA.** (A) Experimental schedule for *in vivo* biosafety evaluation. (B) Representative Masson’s trichrome staining images of superior rectus muscles collected at 7 and 30 days after injection. Scale bar: 200 μm. (C) Quantitative analysis of intraocular pressure (IOP) measurements before and after injection (mean ± SD, *n* = 3). (D) Quantitative analysis of blood biochemistry of ALT, AST, BUN, CREA, CK, and LDH (mean ± SD, *n* = 3).

To assess the local and systemic biosafety of **Gel Nap+BTXA**, Masson’s trichrome staining, IOP monitoring, and blood biochemical analyses were performed (**Figure 7A**). Masson’s trichrome staining showed no noticeable collagen deposition in the superior rectus muscle sections from both the BTXA and **Gel Nap+BTXA** groups, as in the PBS control group (**Figure 7B**). Immunohistochemical staining results of CD45 and CD68 expression in superior rectus muscle sections also showed no obvious local inflammatory response among the three groups (**Figure S19**). In addition, the results of IOP measurement demonstrated that all groups maintained normal values without significant fluctuation, indicating that neither BTXA nor **Gel Nap+BTXA** affected aqueous humor dynamics or ocular physiology (**Figure 7C**). Serum biochemical analyses further revealed no abnormalities in hepatic (ALT, AST) or renal (BUN, CREA) function, nor in muscle injury markers (CK, LDH), suggesting the absence of systemic toxicity (**Figure 7D**). Collectively, the above results demonstrated the excellent biosafety of **Gel Nap+BTXA** *in vivo*.

## Conclusions

In the current research, we developed a supramolecular peptide hydrogel **Gel Nap+BTXA** for sustained and localized delivery of BTXA in extraocular muscles for strabismus treatment. The hydrogel was facilely prepared by co-assembly of hydrogelator NapFFKKK with BTXA under mild physiological conditions. *In vitro* characterizations showed that **Gel Nap+BTXA** maintained a uniform nanofibrous architecture, favorable viscoelasticity, and excellent biocompatibility, enabling stable encapsulation, local administration, and controlled release of BTXA. *In vivo* SPECT imaging revealed that, compared with free BTXA solution, **Gel Nap+BTXA** achieved about 3.1-fold higher local drug retention at 24 h post-injection. Consequently, **Gel Nap+BTXA** effectively restricted toxin diffusion and reduced off-target complications such as ptosis. Notably, **Gel Nap+BTXA** also exhibited a more pronounced and longer-lasting pharmacologic effect up to 2 months, allowing comparable therapeutic outcomes with reduced drug dosage and injection frequency. Overall, this study offered a safe and efficient localized delivery strategy for BTXA, addressing key limitations of BTXA in strabismus therapy and holding potential for broad applications in other medical indications requiring precise botulinum toxin administration.

## Supplementary Material

Supplementary methods, figures and table.

## Data Availability

All data of this study are available within the article and supplementary files. Corresponding authors can be contacted for additional reasonable requests.

## Abbreviations

BTXA: botulinum toxin type A
SPECT: single-photon emission computed tomography
FDA: Food and Drug Administration
ECM: extracellular matrix
PBS: phosphate-buffered saline
Na_2_CO_3_: sodium carbonate
TEM: transmission electron microscopy
FBS: fetal bovine serum
CCK-8: cell counting kit-8
TLC: thin-layer chromatography
CPM: counts per minute
ROI: region of interest
H&E: hematoxylin and eosin
CSA: cross-sectional area
IOP: intraocular pressure
ALT: alanine aminotransferase
AST: aspartate aminotransferase
BUN: urea nitrogen
CREA: creatinine
CK: creatine kinase
LDH: lactate dehydrogenase
SD: standard deviation
ANOVA: analysis of variance
HPLC: high-performance liquid chromatography
ESI-MS: electrospray ionization mass spectrometry
NMR: nuclear magnetic resonance
CAC: critical aggregation concentration
