# Supplementary material for: Localized and sustained release of Botulinum Toxin A from supramolecular peptide hydrogel for strabismus treatment
Source: Theranostics. 2026 May 1;16(12):6497–508. doi: 10.7150/thno.128115 (PMC13231837; doi:10.7150/thno.128115)
Supplement: Supplementary file 1 — Supplementary methods, figures and table. [file thnov16p6497s1.pdf]

Supplementary Material

Localized and sustained release of Botulinum Toxin A from supramolecular peptide hydrogel for strabismus treatment

Wei Guo[1*], Hui Zhu[1*], Yuchen Yao[2], Dan Huang[1], Tiantian Zhou[1], Xiaoqi Zhu[1], Jiaying Zhang[2], Gaolin Liang[2✉], Wenjun Zhan[2✉], and Hu Liu[1✉]

1. Department of Ophthalmology, The First Affiliated Hospital with Nanjing Medical University, Nanjing 210029, China

2. State Key Laboratory of Digital Medical Engineering, School of Biological Science and Medical Engineering, Southeast University, Nanjing 211189, China

* These authors contributed equally to this work.

✉ Corresponding authors: gliang@seu.edu.cn (G.L.), wjzhan@seu.edu.cn (W.Z.), liuhu@njmu.edu.cn (H.L.).

Contents:

1. General methods

2. Synthesis and characterizations of Nap-Phe-Phe-Lys-Lys- Lys (NapFFKKK)

3. Supporting figures and tables

1. General methods

Materials. All reagents were of analytical grade and used without further purification unless otherwise specified. 2-Chlorotrityl chloride resin, 2-naphthylacetic acid (Nap), Fmoc-protected amino acids, HBTU, HOBt, DIPEA, DMF, isopropanol, n-hexane, and TFA were purchased from Macklin Biochemical (Shanghai, China), Aladdin Chemistry (Shanghai, China), Sigma-Aldrich (Shanghai, China), and GL Biochem (Shanghai, China). Botulinum toxin type A (BTXA, Hengli®) was purchased from Lanzhou Biotechnique Development (Lanzhou, China). SNAP-25 polyclonal antibody was purchased from Proteintech (Wuhan, China). Vimentin monoclonal antibody was purchased from Proteintech (Wuhan, China). DAB detection kit was purchased from Maixin Biotechnology (Fuzhou, China). Cell Counting Kit-8 (CCK-8) was obtained from Dojindo Laboratories (Kumamoto, Japan). Annexin V-FITC/PI Apoptosis Kit was purchased from Multi Sciences (Hangzhou, China). FITC-Dextran (MW 150kDa) was purchased from MedChemExpress (Shanghai, China). H&E staining kit, Masson's trichrome staining kit were obtained from Beyotime Biotechnology (Shanghai, China). CD45 and CD68 primary antibodies were purchased from AiFang Biological (Hunan, China). Primary human extraocular muscle fibroblasts were cultured from resected muscle tissues obtained from strabismus surgery with informed consent and institutional ethical approval (Ethics No. 2019-SR-134).

Instruments. High-performance liquid chromatography (HPLC) purification was carried out using a Shimadzu UFLC system equipped with dual LC-20AP pumps and an SPD-20A UV/vis detector, utilizing a Shimadzu PRC-ODS column. Electrospray

ionization mass spectrometry (ESI-MS) analysis was performed on a Finnigan LCQ Advantage ion trap mass spectrometer (Thermo Fisher Scientific, USA). ^{1}H nuclear magnetic resonance (NMR) and ^{13}C-NMR spectra were recorded on a Bruker AV 300. Transmission electron microscopy (TEM) observations were conducted using a Tecnai G2 microscope (FEI, USA). Rheology tests were evaluated with a rheometer (HAAKE RheoStress 6000, Thermo Scientific, Germany). *Ex vivo* fluorescence images were acquired using an IVIS Lumina III system (Revvity, USA). SPECT imaging was conducted on a Discovery NM/CT 670 system (GE Healthcare, USA). The radioactivity (counts per minute, CPM) of the samples was quantified using an automatic gamma counter (Wizar^2 2470, PerkinElmer, USA). Intraocular pressure (IOP) was measured with a handheld rebound tonometer (SW-500, SuoWei, China). Blood biochemistry was analyzed using an automatic biochemical analyzer (Chemray 800, China).

Critical aggregation concentration (CAC). The CAC of NapFFKKK was determined by transmittance analysis. Solutions with concentrations ranging from 10 μM to 5 mM were prepared in PBS of pH 6.5 and pH 7.8, respectively. The transmittance at 650 nm was measured to calculate the CAC value under different pH conditions.

Cytotoxicity assay. CCK-8 assay was used to assess cell viability in response to **Gel Nap** exposure. A total of 5×10^3 cells were seeded per well in 96-well plates and incubated for 24 h to allow cell attachment prior to exposure. The culture medium was then exchanged for fresh medium with different concentrations of **Gel Nap** (0.1, 0.25, 0.5, and 1.0 mg/mL). Following a further 24 h treatment, 200 μL of fresh culture

medium supplemented with 10% CCK-8 reagent was added to each well, and the plates were incubated for additional 2 h. The absorbance at 450 nm was subsequently measured and cell viability (%) was determined as: (ODsample - ODblank) / (ODcontrol - ODblank) × 100%.

Hemolysis assay. Fresh rabbit blood was centrifuged at 3000 rpm for 5 min, and the collected erythrocytes were washed for three times with PBS before being diluted to prepare a 5% suspension. Equal volumes of erythrocyte suspension and different concentrations of **Gel Nap** (0.1-1.0 mg/mL) were mixed and incubated at 37 °C for 3 h. For controls, PBS was utilized as negative control, and 1% Triton X-100 as positive control. After centrifugation (800 rpm, 15 min), the absorbance of the supernatant was recorded at 570 nm, and the hemolysis ratio (%) was calculated as: (ODsample - ODnegative) / (ODpositive - ODnegative) × 100%.

Western blot analysis of SNAP-25. Neuro-2a cells were cultured in reduced-serum medium for differentiation and then treated with PBS, BTXA, or **Gel Nap+BTXA** for 24 h. After treatment, cells were collected for total protein extraction. Equal amounts of protein were separated by SDS-PAGE and transferred onto PVDF membranes. The membranes were blocked and incubated with anti-SNAP-25 and anti-β-actin antibodies. After washing, the membranes were incubated with HRP-conjugated secondary antibodies. Protein bands were detected using chemiluminescence, and band intensities were quantified by densitometric analysis.

Cell apoptosis assay. Flow cytometry was employed to evaluate apoptosis in

fibroblasts. Cells were plated in 6-well culture plates at a density of 2×10^5 cells per well and exposed to **Gel Nap** at final concentrations of 0.1, 0.25, 0.5, and 1.0 mg/mL for 24 h. Following treatment, cells were harvested, rinsed for three times with ice-cold PBS, and resuspended in binding buffer. Apoptotic staining was performed by adding Annexin V-FITC (5 μL) and PI (10 μL), followed by incubation for 15 min at room temperature in the absence of light. Flow cytometric analysis was subsequently conducted, and the proportion of alive cells was quantified.

***Ex vivo* fluorescence imaging.** FITC-Dextran (MW 150 kDa) and BTXA were separately dissolved in PBS to final concentrations of 0.5 mg/mL and 100 U/mL, respectively. Equal volumes of the two solutions were mixed thoroughly prior to injection. A total volume of 0.1 mL of the mixture was then injected into the right superior rectus muscle of the rabbit following the same surgical procedure. Animals were euthanized immediately after injection, and the injected superior rectus muscle, peri-injection tissue (including conjunctiva and Tenon's capsule), as well as the medial rectus and lateral rectus were carefully excised. *Ex vivo* fluorescence imaging was performed in three rabbits, followed by quantitative analysis of the fluorescence intensity.

Immunohistochemical staining. Superior rectus muscle tissues collected at 7 and 30 days after injection were used for immunohistochemical staining of CD45 and CD68. The primary antibodies were used at dilutions of 1:500 and 1:600, respectively. Immunoreactivity was visualized with a DAB detection kit. The stained sections were

then observed and photographed under a light microscope.

Animals. All animal experiments were approved by the Institutional Animal Care and Use Committee of Nanjing Medical University (Approval No. 2409058). Male New Zealand white rabbits were obtained from Pizhou Dongfang Breeding (Xuzhou, China).

2. Syntheses and Characterizations of Nap-Phe-Phe-Lys-Lys-Lys (NapFFKKK)

Scheme S1. Synthetic route of **NapFFKKK**.

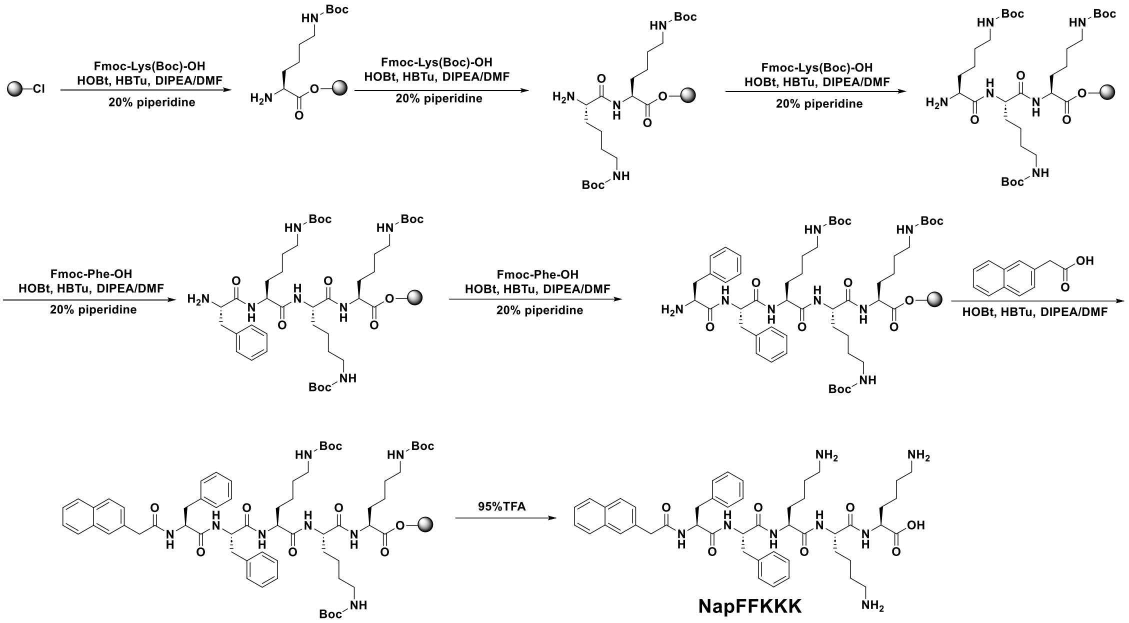

Synthesis of **NapFFKKK**: Compound Nap-Phe-Phe-Lys-Lys-Lys (**NapFFKKK**) was prepared by Fmoc-based solid-phase peptide synthesis (SPPS) on 2-chlorotrityl chloride resin. Briefly, Fmoc-protected amino acids (Fmoc-Phe-OH, Fmoc-Lys(Boc)-OH) were sequentially coupled in DMF using HBTU/HOBt/DIPEA as the coupling system after Fmoc removal with 20% piperidine. After chain assembly, Nap was introduced as the N-terminal capping group. The peptide was subsequently cleaved from the resin using 95% TFA through two successive 30-minute cycles and finally purified by HPLC.

MS: calculated for **NapFFKKK** $[M + H]^+$: 865.5; obsvd. ESI-MS (*m*/*z*) $[M + H]^+$: 865.6 (**Figure S1**). ^{1}H NMR (600 MHz, DMSO-d_6) δ 8.38 (d, *J* = 8.4 Hz, 1H), 8.21 (d, *J* = 8.0 Hz, 1H), 8.18 (d, *J* = 7.8 Hz, 1H), 8.15 (d, *J* = 7.4 Hz, 1H), 8.07 (d, *J* = 7.7 Hz, 1H), 7.85 (d, *J* = 7.8 Hz, 1H), 7.78 (d, *J* = 7.9 Hz, 1H), 7.74 (d, *J* = 8.5 Hz, 1H), 7.58 (s, 1H), 7.50 – 7.44 (m, 2H), 7.26 – 7.20 (m, 4H), 7.20 – 7.12 (m, 7H), 4.57 (td, *J* = 8.6,

4.5 Hz, 1H), 4.50 (td, J = 9.9, 4.1 Hz, 1H), 4.29 (dq, J = 13.4, 8.4, 6.9 Hz, 2H), 4.13 (q, J = 8.1 Hz, 1H), 3.59 (d, J = 14.1 Hz, 1H), 3.49 (d, J = 14.1 Hz, 1H), 3.05 (dd, J = 13.9, 3.8 Hz, 1H), 2.95 (dd, J = 13.7, 3.8 Hz, 1H), 2.86 – 2.79 (m, 1H), 2.75 (q, J = 6.9 Hz, 6H), 2.71 (d, J = 13.7 Hz, 1H), 1.74 – 1.65 (m, 3H), 1.57 (dt, J = 27.9, 6.6 Hz, 9H), 1.34 (dt, J = 15.4, 7.7 Hz, 6H) (**Figure S2**). ^{13}C NMR (151 MHz, DMSO-d_6) δ 173.52, 171.58, 171.34, 171.28, 170.93, 170.04, 137.83, 137.69, 133.91, 132.97, 131.79, 129.33, 129.28, 128.11, 128.00, 127.64, 127.53, 127.50, 127.43, 127.31, 126.35, 126.24, 126.05, 125.53, 54.02, 53.84, 52.40, 52.27, 51.97, 42.27, 38.75, 38.74, 38.65, 37.44, 37.41, 31.54, 31.52, 30.54, 26.73, 26.66, 22.44, 22.30, 22.22 (**Figure S3**).

3. Supporting Figures and Tables

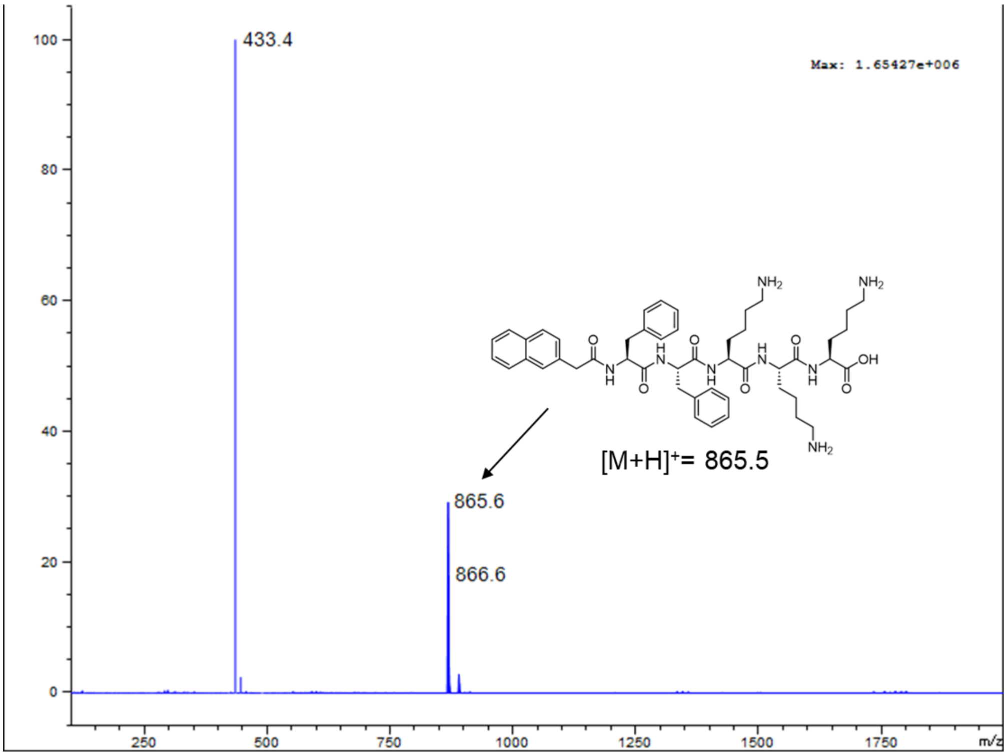

Figure S1. ESI-MS spectrum of **NapFFKKK**.

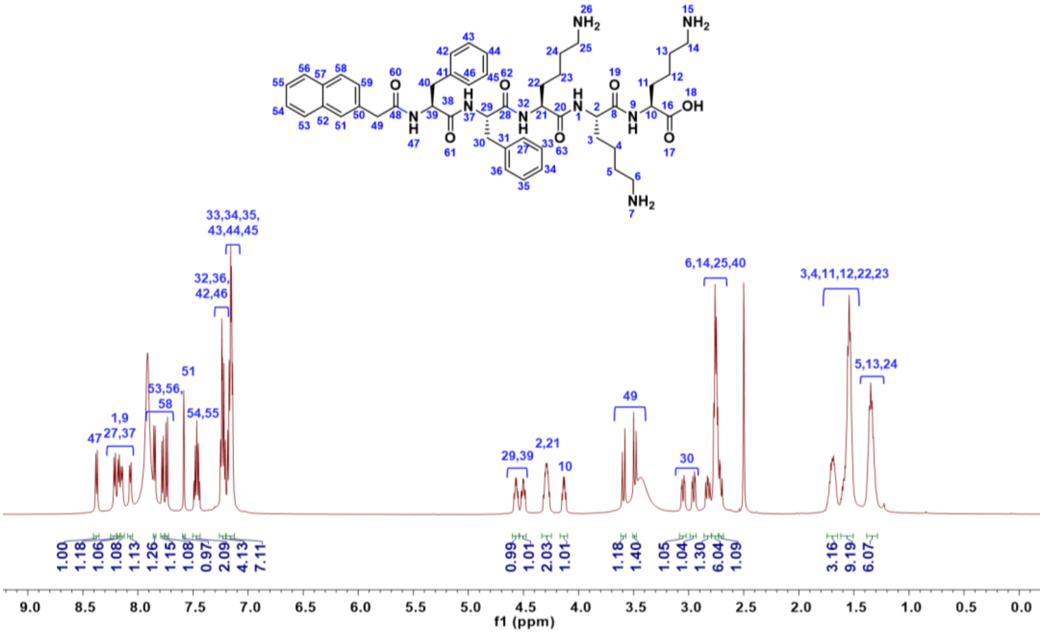

Figure S2. ^{1}H NMR spectrum of **NapFFKKK** in DMSO-*d6*.

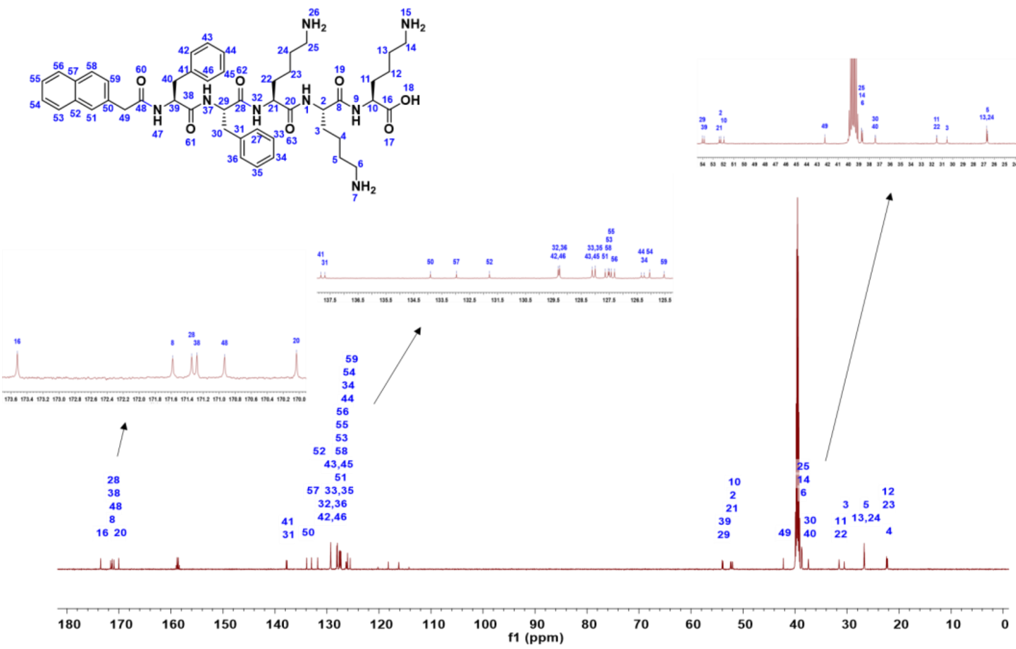

Figure S3. ^{13}C NMR spectrum of **NapFFKKK** in DMSO-*d6*.

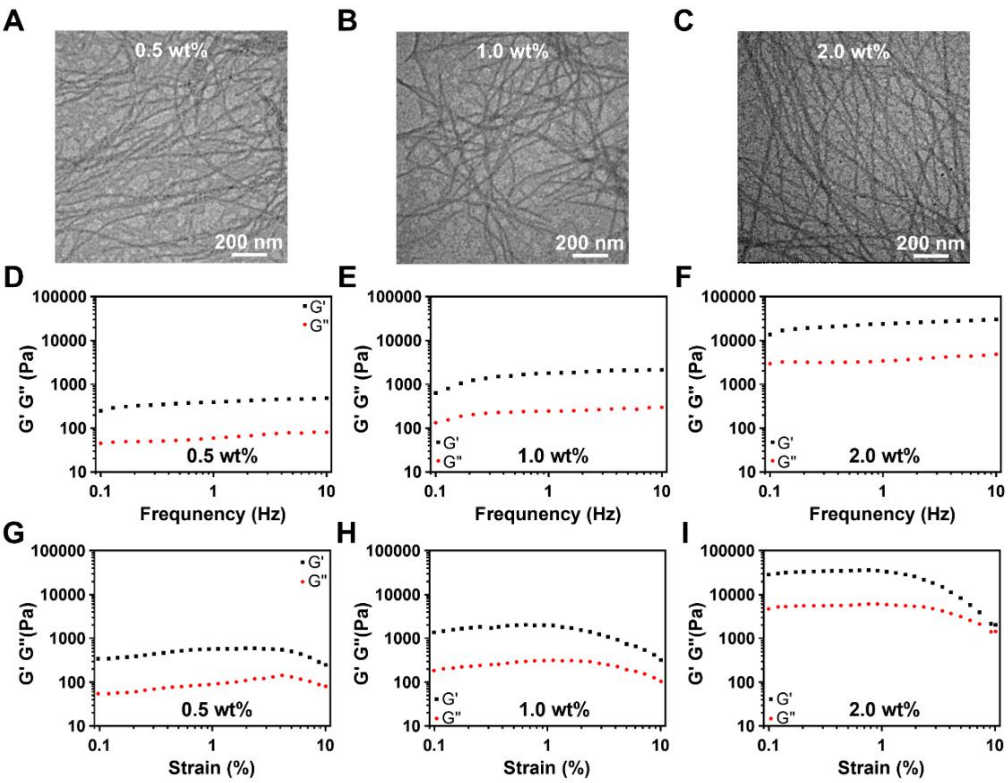

Figure S4. Characterizations of Gel NapFFKKK at different peptide concentrations. (A-C) TEM images of Gel NapFFKKK prepared at 0.5, 1.0, and 2.0 wt%. Scale bar: 200 nm. (D-I) Frequency ($\gamma = 1\%$) and strain ($f = 1$ Hz)-dependent rheology of Gel NapFFKKK prepared at 0.5, 1.0, and 2.0 wt%.

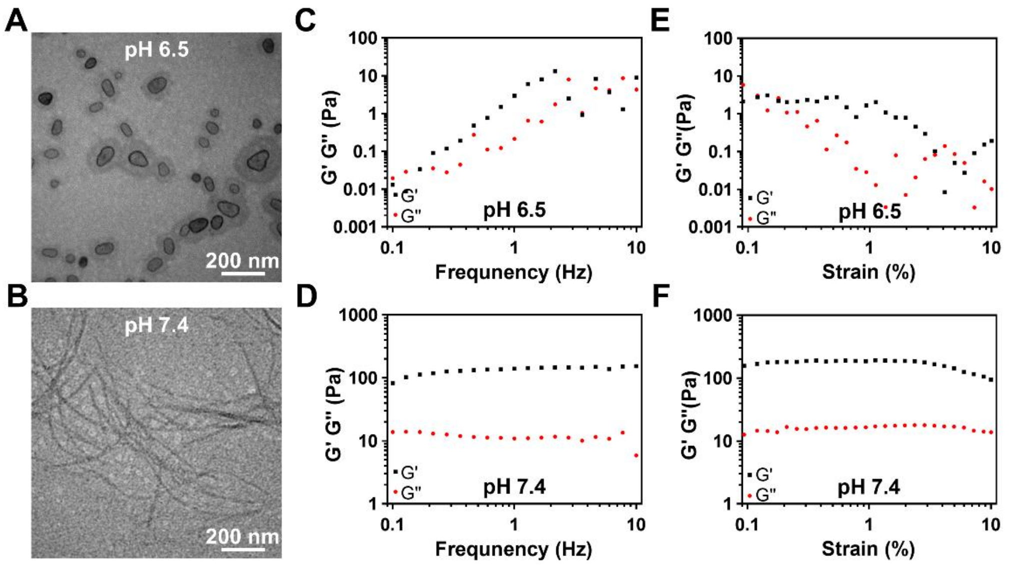

Figure S5. Microstructure and rheology of NapFFKKK under different pH conditions. (A-B) TEM images of NapFFKKK samples prepared at pH 6.5 and 7.4. Scale bar: 200 nm. (C-F) Frequency (γ = 1%) and strain (f = 1 Hz)-dependent rheology of NapFFKKK samples prepared at pH 6.5 and 7.4.

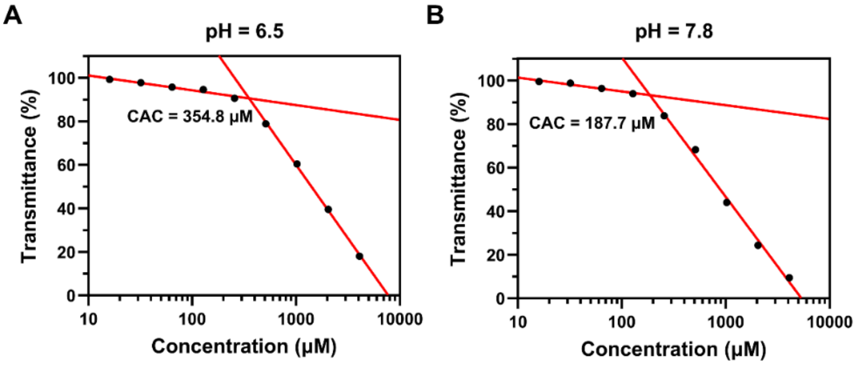

Figure S6. Critical aggregation concentration (CAC) measurement of **NapFFKKK**.

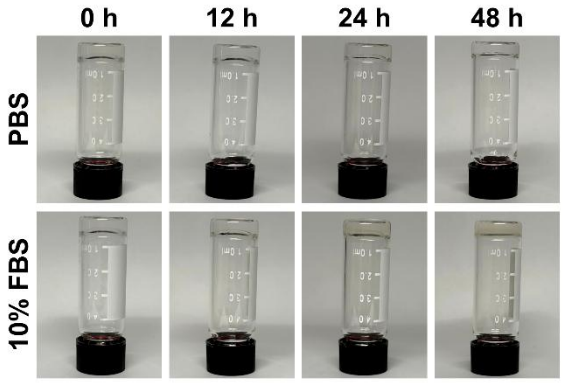

Figure S7. Photographs of **Gel Nap** incubated with PBS (pH 7.4) or 10% FBS at 37 °C for 0, 12, 24, and 48 h.

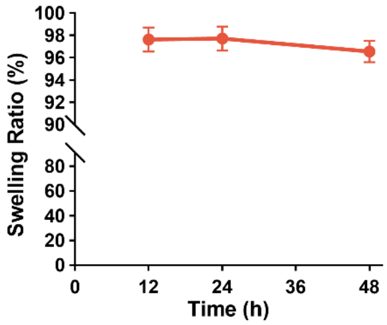

Figure S8. Swelling test of **Gel Nap** incubated with PBS (pH 7.4) for 12, 24, and 48 h.

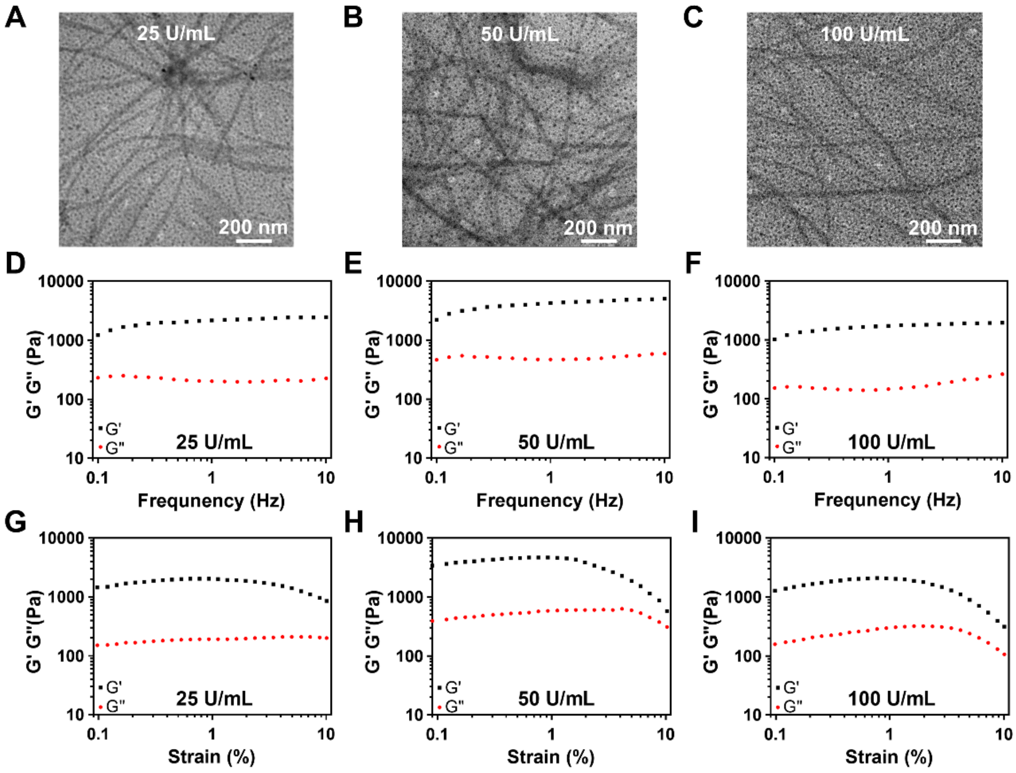

Figure S9. Characterizations of **Gel Nap+BTXA** with different BTXA concentrations. (A-C) TEM images of **Gel Nap+BTXA** loaded with 25, 50, and 100 U/mL BTXA. Scale bar: 200 nm. (D-I) Frequency (γ = 1%) and strain (f = 1 Hz)-dependent rheology of **Gel Nap+BTXA** loaded with 25, 50, and 100 U/mL BTXA.

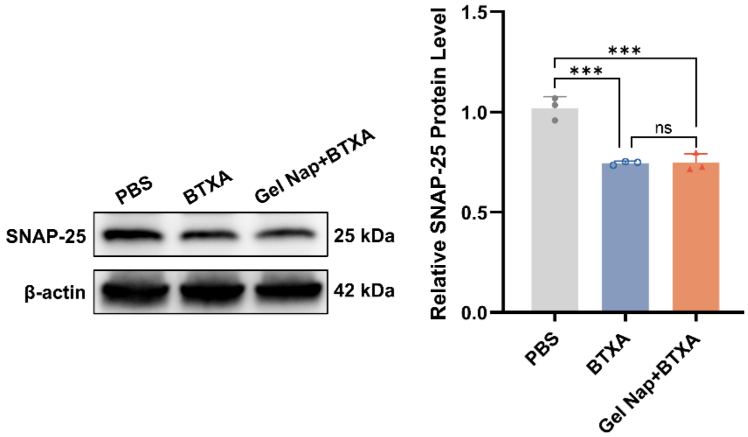

Figure S10. Western blot analysis of SNAP-25 protein in Neuro-2a cells treated with PBS, BTXA, and **Gel Nap+BTXA** (mean ± SD, n = 3, ***p < 0.001).

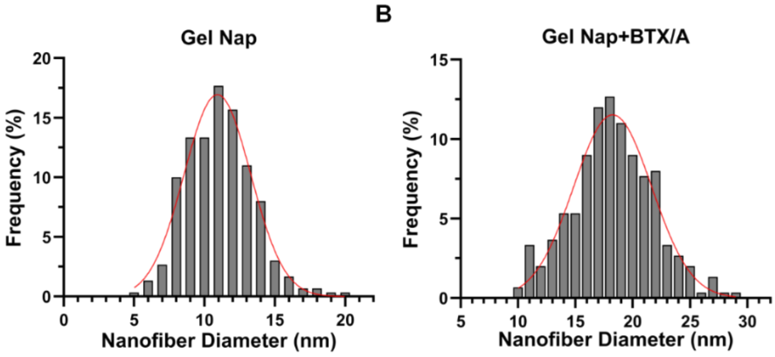

Figure S11. Statistical distribution of nanofiber diameters of **Gel Nap** and **Gel Nap+BTXA** on TEM images in Figure 2B, C.

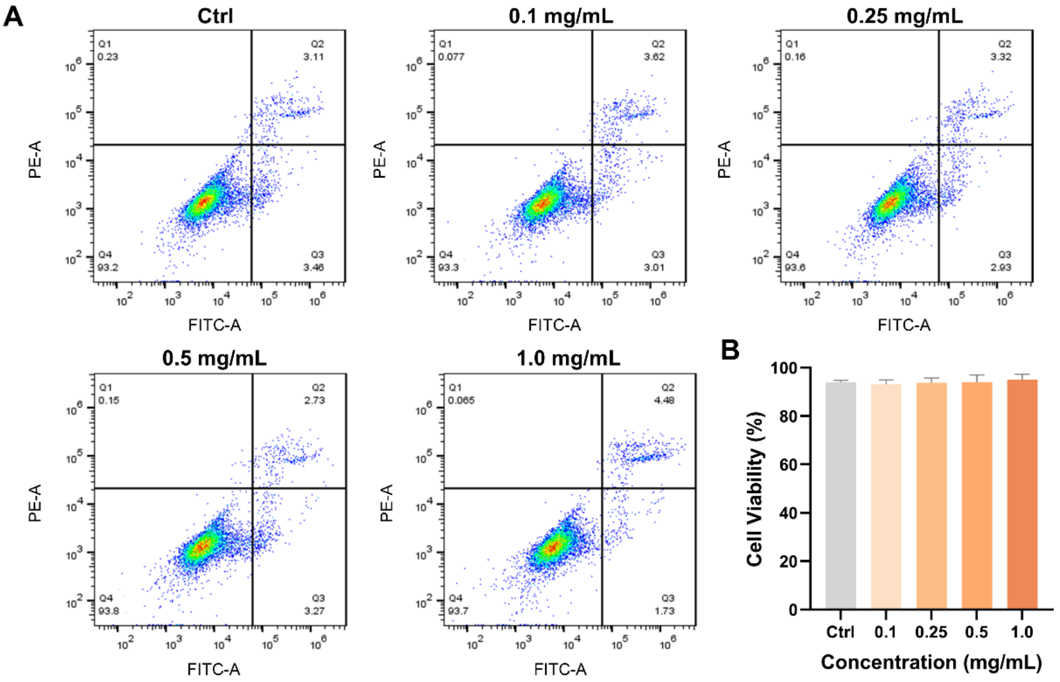

Figure S12. Apoptosis analysis of primary human extraocular muscle fibroblasts. (A) Apoptosis detection of fibroblasts co-incubated with different concentrations of hydrogel. (B) Quantitative analysis of surviving cells after incubation with 0.1-1.0 mg/mL hydrogel (mean ± SD, $n = 3$).

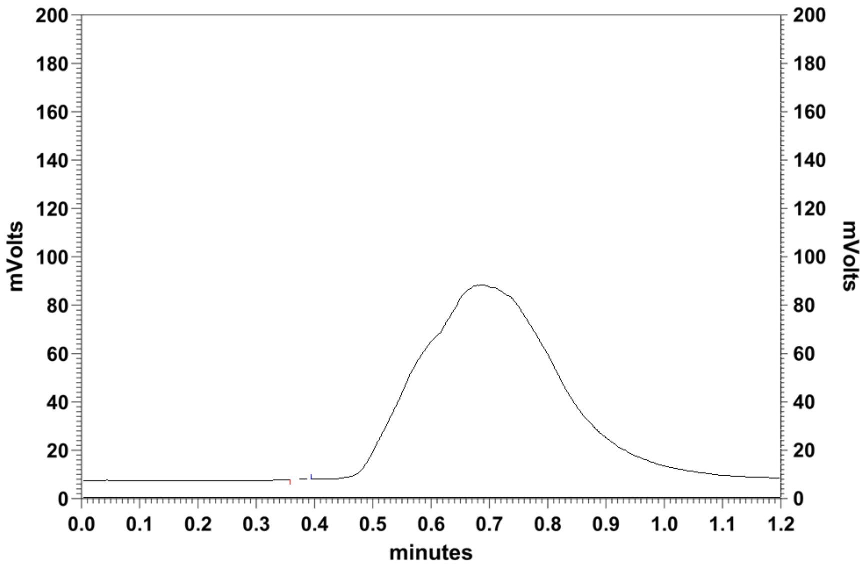

Figure S13. Thin-layer chromatography (TLC) profile of free ^{131}I.

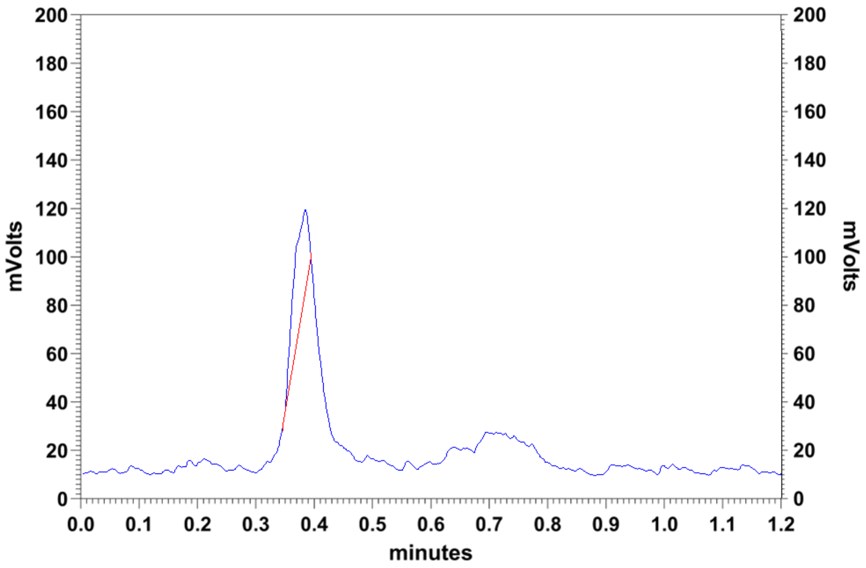

Figure S14. Thin-layer chromatography (TLC) profile of ^{131}I-BTXA.

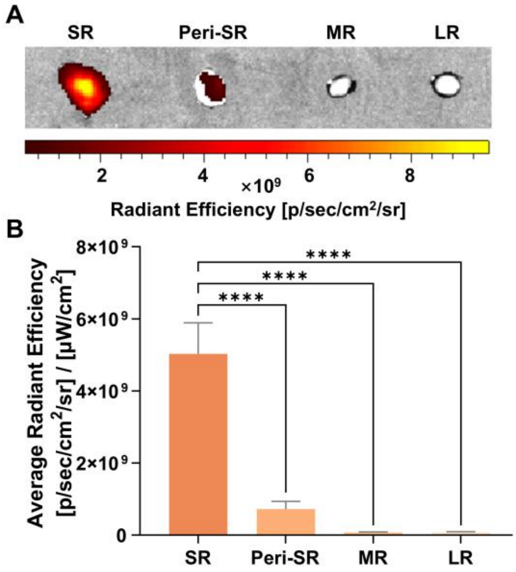

Figure S15. (A) Representative *ex vivo* fluorescence images of the injected superior rectus muscle (SR), adjacent peri-injection tissue, medial rectus, and lateral rectus muscle. (B) Quantification of fluorescence intensities in A (mean ± SD, $n = 3$, ****$p < 0.0001$).

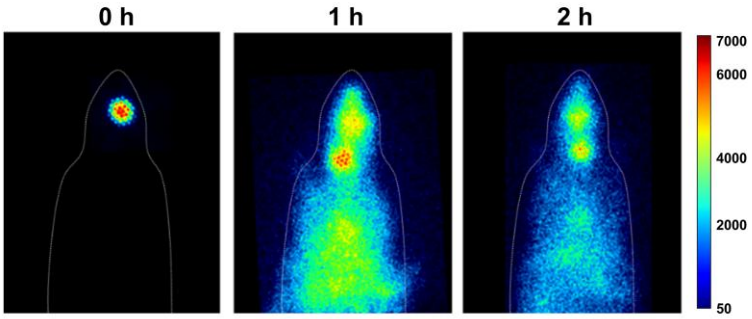

Figure S16. SPECT images at 0 h, 1 h, 2 h after intramuscular injection of free ^{131}I in the superior rectus muscle.

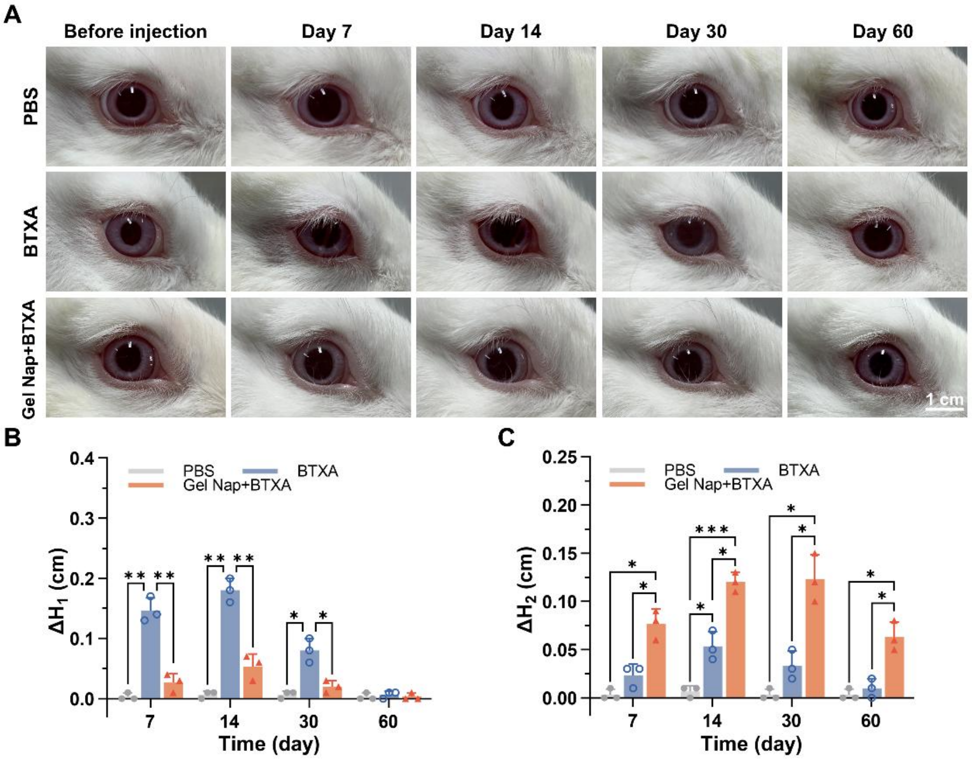

Figure S17. (A) Representative photographs of the injection eye in the PBS, BTXA (2.5 U), and **Gel Nap+BTXA** (2.5 U BTXA) groups at 7, 14, 30, and 60 days after injection. Scale bar: 1 cm. (B) Quantitative analysis of ptosis (ΔH_1) in the three groups. (C) Quantitative analysis of ocular alignment (ΔH_2) in the three groups (mean ± SD, n = 3, *$p < 0.05$, **$p < 0.01$, ***$p < 0.001$).

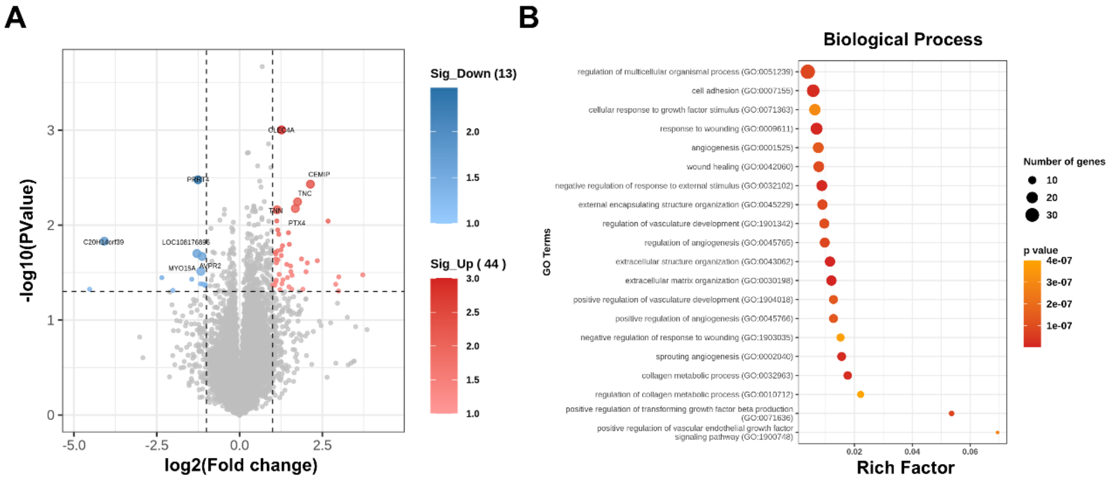

Figure S18. Transcriptomic analysis of extraocular muscle. (A) Volcano plot showing differentially expressed genes between **Gel Nap+BTXA** group and BTXA group. (B) Biological process enrichment analysis based on Gene ontology for upregulated genes.

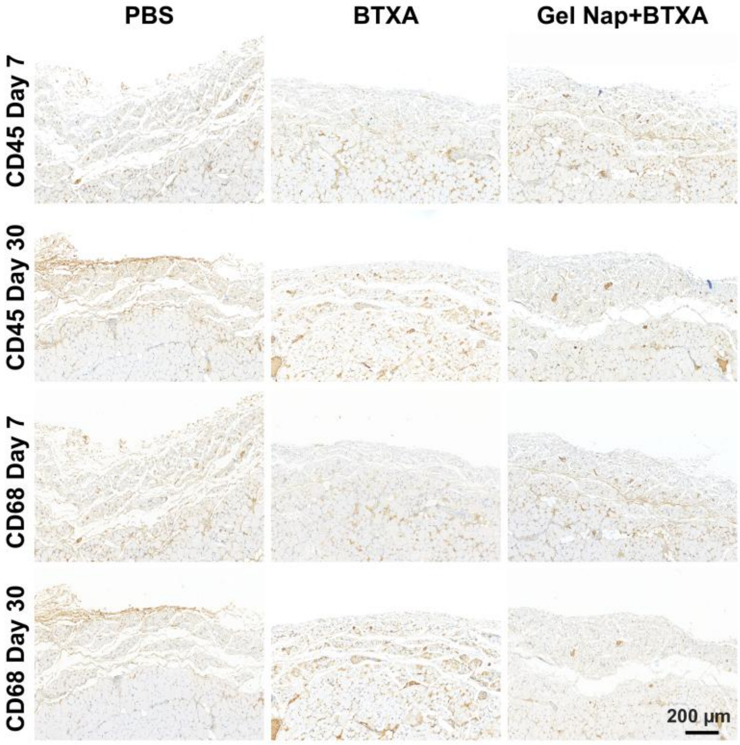

Figure S19. Representative immunohistochemical staining images of CD45 and CD68 expression in superior rectus muscle sections collected at 7 and 30 days after injection in PBS, BTXA, and **Gel Nap+BTXA** groups. Scale bar: 200 μm.

Table S1. Radioactivity per gram of ^{131}I-BTXA in target and non-target extraocular muscles at 24 h post-injection.

**Muscle**	**Gel Nap+^{131}I-BTXA (CPM/g)**	**^{131}I-BTXA (CPM/g)**	***p* value**
**SR**	26502424.24 ± 6019414.90	6519957.26 ± 1027016.49	< 0.01
**IR**	226259.89 ± 15334.49	477108.01 ± 364278.20	0.299
**MR**	446472.06 ± 24176.80	514923.08 ± 482426.31	0.837
**LR**	285000.00 ± 44989.44	731682.54 ± 25010.54	< 0.01
**LPS**	746795.70 ± 55400.55	4200066.08 ± 17971.38	< 0.01

Abbreviations: BTXA: botulinum toxin type A; CPM: counts per minute; SR: superior rectus; IR: inferior rectus; MR: medial rectus; LR: lateral rectus; LPS: levator palpebrae superioris.
